# Perinatal environment shapes microbiota colonization and infant growth: impact on host response and intestinal function

**DOI:** 10.1186/s40168-020-00940-8

**Published:** 2020-11-23

**Authors:** M. Selma-Royo, M. Calatayud Arroyo, I. García-Mantrana, A. Parra-Llorca, R. Escuriet, C. Martínez-Costa, M. C. Collado

**Affiliations:** 1 Institute of Agrochemistry and Food Technology (IATA-CSIC), Spanish National Research council, 46980 Valencia, Spain; 2 Neonatal Research Group, Health Research Institute La Fe, Valencia, Spain; 3 Gerencia de Procesos Integrales de Salud. Area Asistencial, Servicio Catalan de la Salud. Generalitat de Catalunya, Centre for Research in Health and Economics, Universidad Pompeu Fabra, Barcelona, Spain; 4 Department of Pediatrics, School of Medicine, University of Valencia, 46010 Valencia, Spain; 5 Pediatric Gastroenterology and Nutrition Section, Hospital Clínico Universitario Valencia, INCLIVA, 46010 Valencia, Spain

**Keywords:** Microbiota, Environment, Mode of birth, Antibiotics, Epithelial barrier, Immune system, Early programming

## Abstract

**Background:**

Early microbial colonization triggers processes that result in intestinal maturation and immune priming. Perinatal factors, especially those associated with birth, including both mode and place of delivery are critical to shaping the infant gut microbiota with potential health consequences.

**Methods:**

Gut microbiota profile of 180 healthy infants (*n* = 23 born at home and *n* = 157 born in hospital, 41.7% via cesarean section [CS]) was analyzed by 16S rRNA gene sequencing at birth, 7 days, and 1 month of life. Breastfeeding habits and infant clinical data, including length, weight, and antibiotic exposure, were collected up to 18 months of life. Long-term personalized in vitro models of the intestinal epithelium and innate immune system were used to assess the link between gut microbiota composition, intestinal function, and immune response.

**Results:**

Microbiota profiles were shaped by the place and mode of delivery, and they had a distinct biological impact on the immune response and intestinal function in epithelial/immune cell models. Bacteroidetes and *Bifidobacterium* genus were decreased in C-section infants, who showed higher *z*-scores BMI and W/L during the first 18 months of life. Intestinal simulated epithelium had a stronger epithelial barrier function and intestinal maturation, alongside a higher immunological response (TLR4 route activation and pro-inflammatory cytokine release), when exposed to home-birth fecal supernatants, compared with CS. Distinct host response could be associated with different microbiota profiles.

**Conclusions:**

Mode and place of birth influence the neonatal gut microbiota, likely shaping its interplay with the host through the maturation of the intestinal epithelium, regulation of the intestinal epithelial barrier, and control of the innate immune system during early life, which can affect the phenotypic responses linked to metabolic processes in infants.

**Trial registration:**

NCT03552939.

Video Abstract

**Supplementary Information:**

The online version contains supplementary material available at 10.1186/s40168-020-00940-8.

## Introduction

Microbial colonization plays an important role in numerous functions, including digestion, metabolic reactions, and trophic effects, and it also influences the development and maturation of the host’s innate and adaptive immune system [1–3]. The mode of birth is a key factor shaping early microbial colonization [4–6]. Vaginally born (VAG) infants acquire microbial communities resembling the maternal vaginal and gut microbiota, whereas infants born via C-section acquire environmental-like bacteria such as *Staphylococcus*, *Corynebacterium*, and *Propionibacterium* spp. [7]. In addition, CSs are associated with lower microbial diversity, delayed colonization of *Bacteroides* and *Bifidobacterium* spp., and reduced immune responses [8]. The CS rate increased by an annual increment of 3.7% from 2000 to 2015 worldwide [9, 10]. In Europe, the average CS rate is 28% [11], although the World Health Organization (WHO) recommends a rate of 10–15% [12]. Epidemiological studies have linked CSs with a higher risk of non-communicable diseases such as obesity [13, 14] and allergy [15]. Indeed, the CS procedure is characterized by pre- and intra-partum antibiotic exposure and other medical practices, which may affect early gut colonization and predispose the infant to developing immune-related disorders later in life, including asthma [16–18], allergy [19–21], obesity [22, 23], and diabetes [24, 25]. Hospital interventions during birth are critical for pioneer microbial colonizers and proper immune system maturation [26, 27], which may impact adult health [28, 29]. However, neonatal microbiota colonization in the absence of hospital interventions remains underexplored. Furthermore, the hospital environment (high-level disinfection and antibiotic therapy) has an impact on microbial exposure, thereby extending the hygiene hypothesis to the time of birth [30, 31].

Home births (HBs) increased by 77% between 2004 and 2017 in the USA, while the rate of birth-center deliveries doubled during the same period [32]; however, in Europe, such births account for less than 1% of all deliveries, except in the Netherlands, where HBs represent 16.3% of births [33]. Recently, a distinct microbiota profile has been reported in VAG neonates born at home or in hospital [34], although the impact on microbiota development and the potential effects on neonatal health are not fully understood.

In this study, we investigate the influence of birth-related factors, including the place and mode of delivery on early colonization during the first month of life and on infant growth during the first 18 months. Furthermore, to understand the potential biological mechanisms involved, in vitro gut models are used to study the impact of distinct microbiota patterns on intestinal function and innate immune system maturation.

## Results

### Study population

No differences were observed in the neonatal weight between groups, which showed a median of 3250 g (range 2973–3573 g). Other maternal clinical parameters are presented in the supplementary material (Additional file 1). Despite all the babies were born at full term, the CS-born neonates were born before the both groups of vaginally delivered neonates (39 weeks of pregnancy for CS births and 40 weeks for the VAG and HB neonates, respectively) (*p = 0*.*004*).

The HB neonates showed higher length measurements than the hospital-born infants for both delivery modes (*p = 0*.*003*). Additionally, the HB infants had higher ratios of exclusive breastfeeding than the hospital-delivered infants at both 7 and 31 days of life (*p < 0*.*001*).

### Perinatal factors related to the place and mode of delivery shape the neonatal microbiome composition at birth

**Fig. 1:**
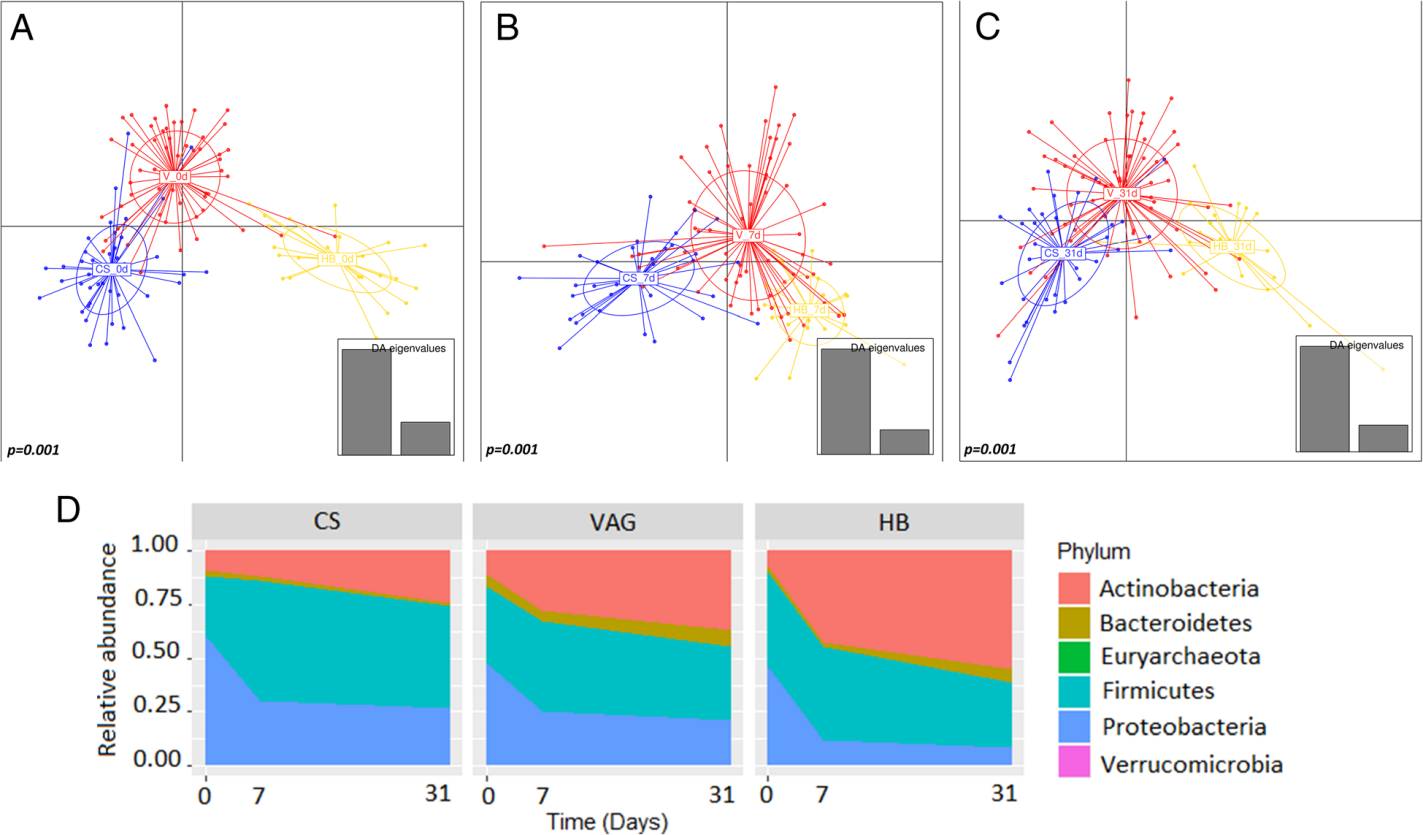
Factors affecting neonatal microbiota during the first month of life. **a**–**c** Discriminant analysis of principal components (DAPC) of the neonatal (**a**) and infant fecal microbiota at 7 days (**b**) and 31 days (**c**) at ASV level. Each point represented microbiota from a neonate. Adonis analysis was used to stablish the significance of studied variables. **d** Colonization patterns during the first moth of life. Neonatal microbiota composition at phylum level at birth (0d), 7 days (7d), and 1 month (31d). C-section (CS, *n* = 65), vaginal delivery at hospital (VAG, *n* = 92), and homebirth (HB, *n* = 23)

At birth, the place (hospital versus home) was the main contributor of neonatal microbiota composition (*p = 0*.*001*), followed by the mode of birth (*p = 0*.*025*) (Fig. 1a). Other perinatal factors did not significantly influence the neonatal microbiota during delivery.

The place and mode of birth shaped the microbial richness and diversity at amplicon sequence variant (ASV) level (Additional file 2). The hospital-born neonates showed a bacterial community with greater richness (*p = 0*.*002*) and diversity (*p = 0*.*072*) at birth than the HB neonates. The CS-born neonates harbored higher index of observed species (*p = 0*.*023*), diversity (*p = 0*.*001*), and richness (*p = 0*.*031*) than the HB infants.

The neonatal fecal microbiota was dominated by the Proteobacteria phylum, followed by Firmicutes and Actinobacteria (Additional file 3). The hospital-born infants showed higher relative abundances of the *Finegoldia* (*p < 0*.*001*), *Clostridioides* (*p < 0*.*001*), *Klebsiella* (*p = 0*.*025*), and *Peptoniphilus* (*p = 0*.*006*) genera, including *Clostridioides difficile* (*p < 0*.*001*) and *Clostridium neonatale* (*p = 0*.*006*), while the HB infants showed higher relative abundances of the *Staphylococcus* (*p < 0*.*007*) and *Enterococcus* (*p < 0*.*001*) genera.

As for the mode of birth, the VAG infants showed higher relative abundances of the Bacteroidetes (*p = 0*.*035*) and Firmicutes (*p = 0*.*035*) phyla, including the *Bacteroides* and *Escherichia/Shigella* genera. A linear discriminant analysis of effect size (LEfSe) was used to confirm which genera were responsible for the clustering of the rectal swab microbial populations (Additional file 4). The microbiota of the HB neonates was enriched by species from the *Enterococcus* and *Staphylococcus* genera, while the hospital-born infants were enriched with species from the *Klebsiella*, *Veillonella*, and *Clostridioides* genus. We also observed a microbial core at each time point and noted some unique genera not shared between the groups (Additional file 5). For instance, we observed that the *Akkermansia* genus was only present in the HB.

### Perinatal factors related to the place and mode of delivery shape neonatal microbiota development

Mode of birth (*p = 0*.*001*) and time (*p = 0*.*001*) were the main contributors to the overall microbiota structure during the first month of life at ASV level. Generally, neonatal microbiota at birth showed significantly higher microbial richness (Chao1 index) and diversity (Shannon index) than the microbiota at 7 and 31 days of life (Additional file 2). No differences in alpha diversity (Chao 1, Shannon index) were found according to the place or mode of delivery at the 7- or 31-day time points.

Distinct colonization patterns were identified between the hospital born (VAG and CS) and HB neonates (Fig. 1d). The mode of birth shaped the microbiota colonization process at 7 (Adonis *p < 0*.*001*) and 31 days (Adonis *p < 0*.*005*) at ASV level.

The infant microbiota showed differing development in the VAG and CS-delivered neonates at the phylum and family levels (Fig. 1d). The VAG deliveries exhibited a colonization pattern somewhere between the patterns of the CS-born and HB deliveries. The HB and CS-born infants showed significant differences in the Firmicutes phylum at delivery (*p = 0*.*035*) and in the Proteobacteria at 7 days (*p = 0*.*019*), although they did not show differences when compared to the hospital-based VAG infants. The relative abundance of the Actinobacteria phylum increased during neonatal life in the both groups of vaginally (VAG and HB) born infants but not in the CS-born neonates (Additional file 3). In the HB neonates, the Actinobacteria increase occurred from delivery to 31 days, while in the hospital-based VAG neonates, the increase was delayed from 7 to 31 days of life. The Bacteroidetes phylum abundance was slightly higher in the vaginal births (VAG and HB) at 7 (*p = 0*.*014*) and 31 days (*p = 0*.*01*) than in the CS births.

At the phylum level, the vaginal births (VAG and HB) showed higher relative abundances of the Actinobacteria (*p = 0*.*001*) and Bacteroidetes (*p = 0*.*018*) phyla, especially *Bifidobacterium* (*p* = 0.003 at 7 days), when compared with the CS-born infants at both times (7and 31 days). The CS-born infants were enriched with the Firmicutes phylum (*p = 0*.*020*), including *Enterococcus* (*p = 0*.*005* at 7 days) and *Clostridium* (*p = 0*.*039*).

The HB neonates harbored a higher relative abundance of Actinobacteria (*p = 0*.*004* and *p = 0*.*006* at 7 and 31 days, respectively) and a lower relative abundance of Proteobacteria (*p = 0*.*026* and *p = 0*.*008* at 7 and 31 days, respectively) when compared with the hospital-born infants (VAG and CS) at both times. Hospital-born infants had higher relative abundances of the *Klebsiella* species and lower relative abundances of *Bifidobacterium* genus, including *B*. *bifidum* (*p = 0*.*013*), and *Collinsella* genus, including *Collinsella aerofaciens* (*p = 0*.*028* at 31 days), compared with the HB neonates.

**Fig. 2:**
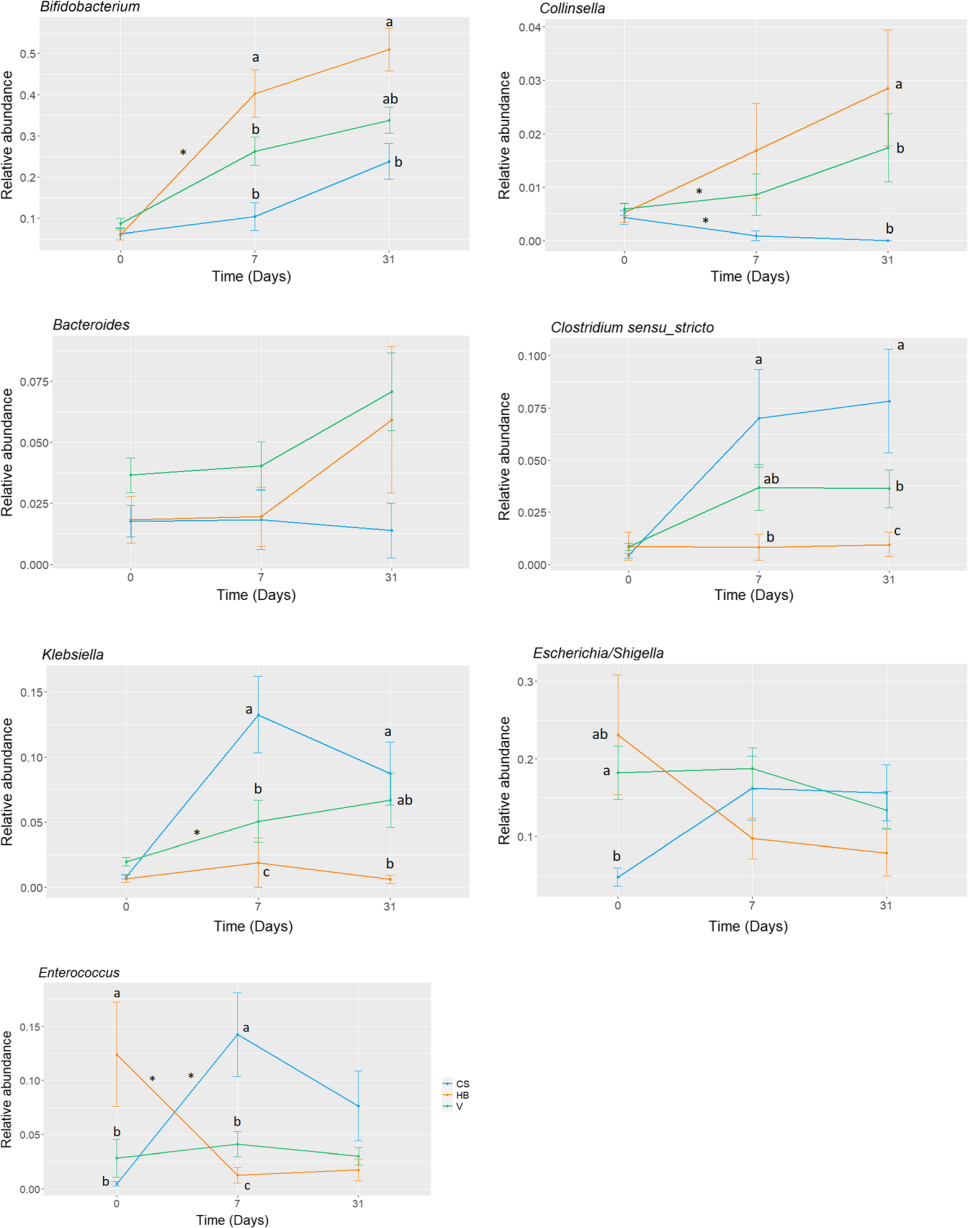
Differences in relative abundance of most important and variable genera in fecal microbiota among the first month of life. Each point represented the mean and SEM of relative abundance of each genus in that point from the fecal samples of babies born by cesarean section (blue), vaginal delivery at hospital (green), and at home (orange). Kruskal-Wallis test with a Dunn’s post hoc test was performed to compare the different groups. Data not sharing the same letter in each point were significantly different (*p* < *0*.*05*). Significant variations within the same group at different time points were marked by an asterisk (*). C-section (CS, *n* = 65), hospitalized vaginal delivery (VAG, *n* = 92), and homebirth (HB, *n* = 23)

In the vaginal births (both VAG and HB), the relative abundances of the *Collinsella* and *Bacteroides* genera increased during the first month of life. An opposite trend was observed for the *Escherichia* and *Enterococcus* genera (Fig. 2). In the CS-born infants, *Escherichia*, *Enterococcus*, and *Klebsiella* genera increased from birth to 7 and 31 days (Fig. 2). The relative abundance of *Bifidobacterium* genus was higher in the vaginal births, especially the HB infants at 7 (*p < 0*.*001*) and 31 days (*p = 0*.*004*), when compared with the CS births. Moreover, CS-neonates harbored a higher relative abundance of the *Clostridium sensu stricto* genus than the HB infants at 7 days (*p = 0*.*007*), although no difference was observed with the hospital-based VAG infants (*p = 0*.*250*), indicating intermediate colonization patterns in neonates born in hospital via vaginal route.

At both 7 and 31 days, the *Bacteroides* genus was present between the vaginal-delivered neonates but not the CS-delivered infants, who showed *Enterobacter* as a unique genus at 31 days Additional file 5.

In a subset of samples, total bacterial counts by qPCR were significantly lower at birth than the counts obtained at 7 days in all three groups (Additional file 6). We also found that the CS-born fecal samples showed significantly lower bacterial counts than the VAG (*p = 0*.*043*) and HB (*p = 0*.*008*) samples at delivery. The HB infants showed higher total bacterial counts than the VAG (*p = 0*.*008*) and CS-born (*p = 0*.*043*) infants at 7 days. Similarly, in the case of the *Bifidobacterium* genus counts in the vaginal-delivered groups (HB [*p < 0*.*001*] and VAG [*p = 0*.*003*]), the counts increased during the first month. Furthermore, the HB infants had higher *Bifidobacterium* counts than the VAG and CS-born infants at 7 (*p = <0*.*001* for CS, *p = 0*.*004* for VAG) and 31 days (*p = 0*.*027* for CS, *p = <0*.*001* for VAG).

### Impact of the perinatal environment on the infant weight status at 18 months

**Fig. 3:**
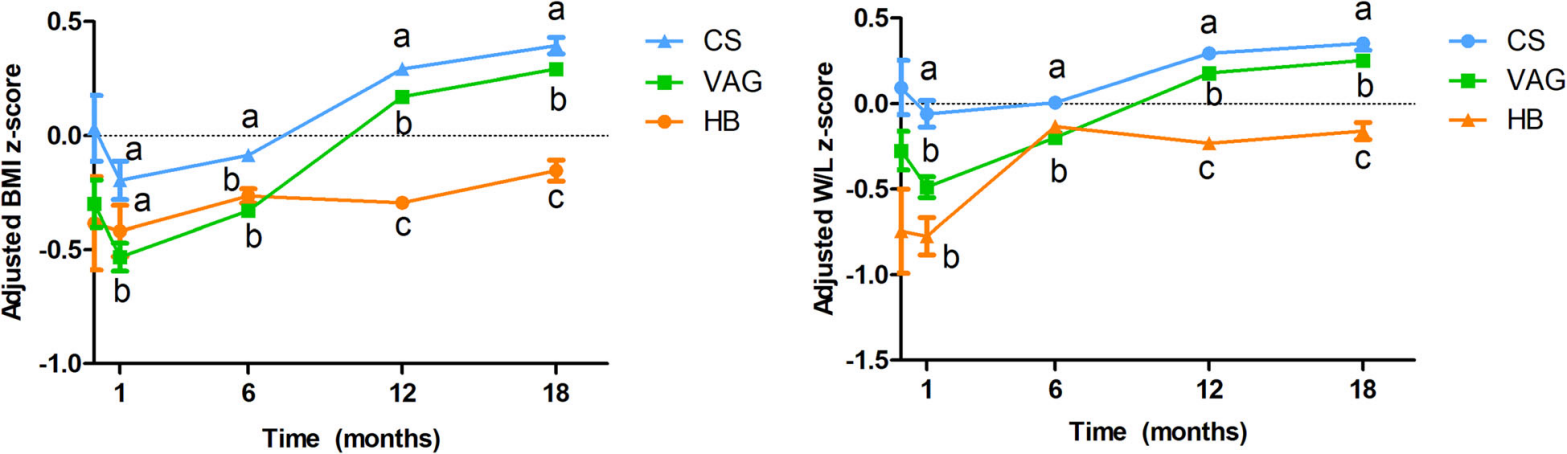
Place and mode of birth impact the infant growth. BMI *z*-scores (**a**) and weight for length (**b**) *z*-scores curves from delivery to 18 months of life according to mode of birth and place adjusted by covariates, breastfeeding duration, antibiotic intake during the first year of life, maternal pre-gestational BMI and infant BMI and weight for length (W/L) *z*-scores at birth. General linear model multivariate test adjusted by covariates was done and *p < 0*.*05* was considered significant. Kruskal-Wallis was performed on the adjusted values (different letters indicate significant differences between three studied groups). C-section (CS, *n* = 58), hospitalized vaginal delivery (VAG, *n* = 85), and homebirth (HB, *n* = 23)

Higher BMI and W/L *z*-scores were observed in the CS-born infants than in the HB (*p = <0.001* for BMI, *p <* *0.001* for W/L at 12 months) and hospital-born vaginal births (*p = <0.001* for BMI and *p =* *0.003* for W/L at 12 months) (Fig. 3a, b). Indeed, at 18 months of life, CS infants also exhibit higher BMI *z*-scores than HB (*p <* *0.001*) and VAG (*p =* *0.016*) children. Additionally, a multivariate linear analysis (adjusted by breastfeeding duration, antibiotic intake during the first year of life, maternal pre-gestational BMI, and BMI and W/L *z*-scores at delivery) showed the CS-born neonates to exhibit significantly higher BMI and W/L *z*-scores across the first 18 months of life.

### Microbiota predicted functionality during the first month of life is influenced by the birth mode and place

**Fig. 4:**
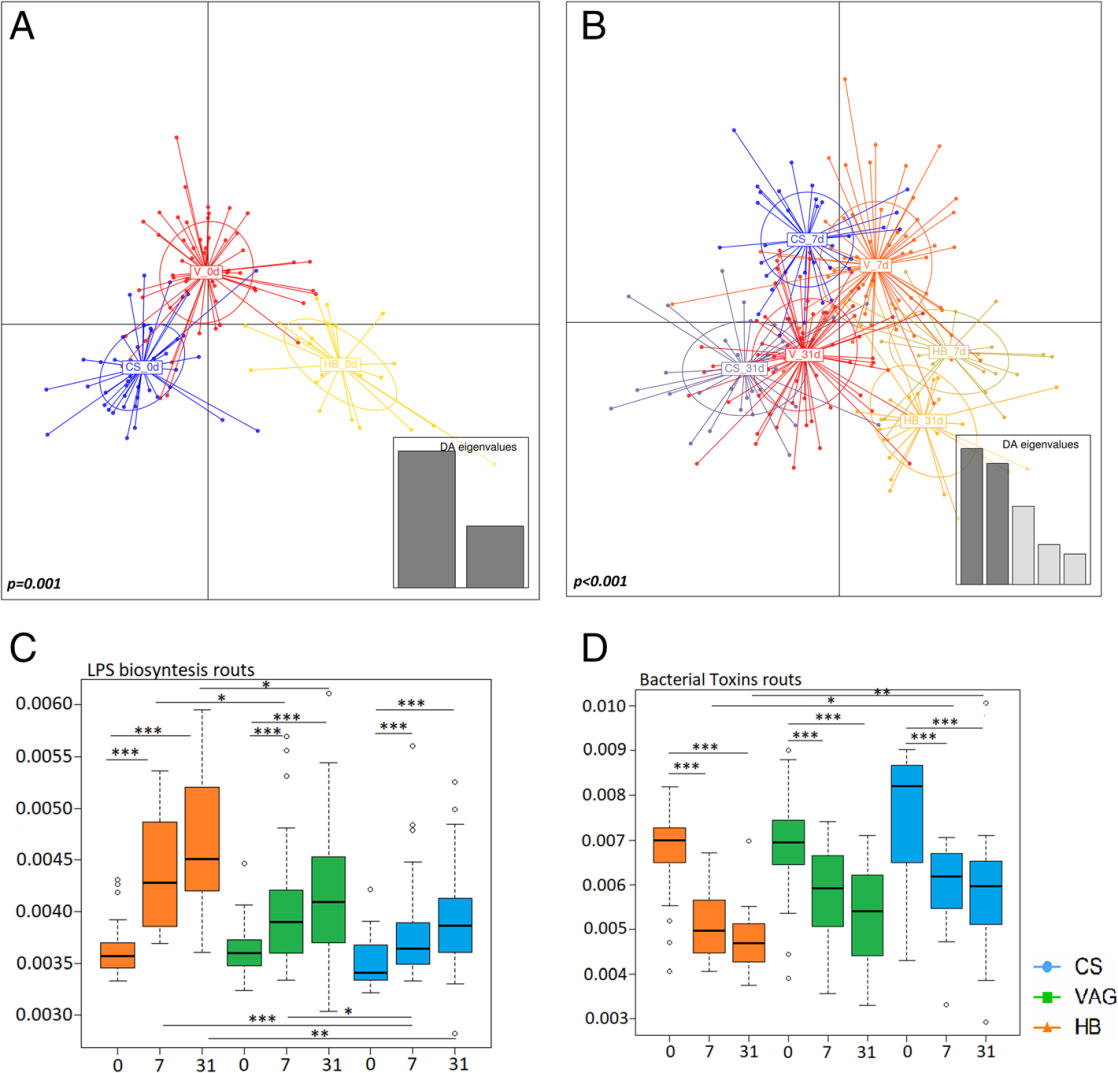
Microbial functions computationally predicted present in neonatal microbiota along the first month of life. **a**, **b** Discriminant analysis of principal components (DAPC) of the neonatal (**a**) and infant fecal microbiota at 7 days and 31 days (**b**). Adonis analysis was used to stablish the significance of studied variables. **c**, **d** Computational analysis of lipopolysaccharide (LPS) biosynthesis (**c**) bacterial toxins (**d**) routs presents in the fecal microbiota of newborns along the first month of life. Results were expressed as percentage of total functional routs for each sample. *p < 0.*05*, **p < 0.*01*, ***p < 0.*001*. C-section (CS, *n* = 65), hospitalized vaginal delivery (VAG, *n* = 92), and homebirth (HB, *n* = 23)

The inferred microbial functionality at birth was mainly affected by the mode of birth (*p < 0*.*001*), but also by the birthplace (*p = 0*.*049*). In addition, mode of birth also influenced the microbiota predicted functionality at 7 (*p = 0*.*001*) and 31 days (*p = 0*.*02*); however, the birthplace was only significant at 7 days (*p = 0*.*001*) (Fig. 4a, b).

Regarding concrete functions, the microbiota of the vaginal-delivered infants (VAG and HB) was enriched in functional routes related to the synthesis of secondary metabolites (*p < 0*.*001*), amino acids (*p = 0*.*002*), lipids (*p = 0*.*034*), and carbohydrate metabolism (*p = 0*.*046*) when compared with the CS-born neonates at delivery (Additional file 7). At 7 and 31 days, the vaginal-delivered infants showed microbiota enriched in lysine, phenylalanine, tyrosine, tryptophan, valine, leucine, and isoleucine biosynthesis pathways. At 1 month of life, the CS-born infants expressed higher energy metabolism pathways, including carbohydrates, lipids, and propanoate biosynthesis.

The immune-system-related paths (e.g., antigen processing and presentation [*p < 0*.*001*] and NOD-like receptors [*p < 0*.*001*]) were overrepresented in the CS-born infants at birth. Contrarily, the lipopolysaccharide (LPS) biosynthesis routes were enriched in the vaginal-delivered infants at all time points (Fig. 4c, d), being also influenced by the birthplace. The genera that mostly contribute to the differences in these predicted routs were *Escherichia/Shigella*, *Klebsiella*, and *Clostridium sensu stricto*. However, the CS-delivered infants showed increasing representation of pathways related to bacterial toxins at 7 (*p = 0*.*011*) and 31 days (*p = 0*.*004*) when compared with the HB neonates.

### Fecal supernatants induce the mRNA expression of *TLR4* and *IRAK4* in intestinal epithelial cells

**Fig. 5:**
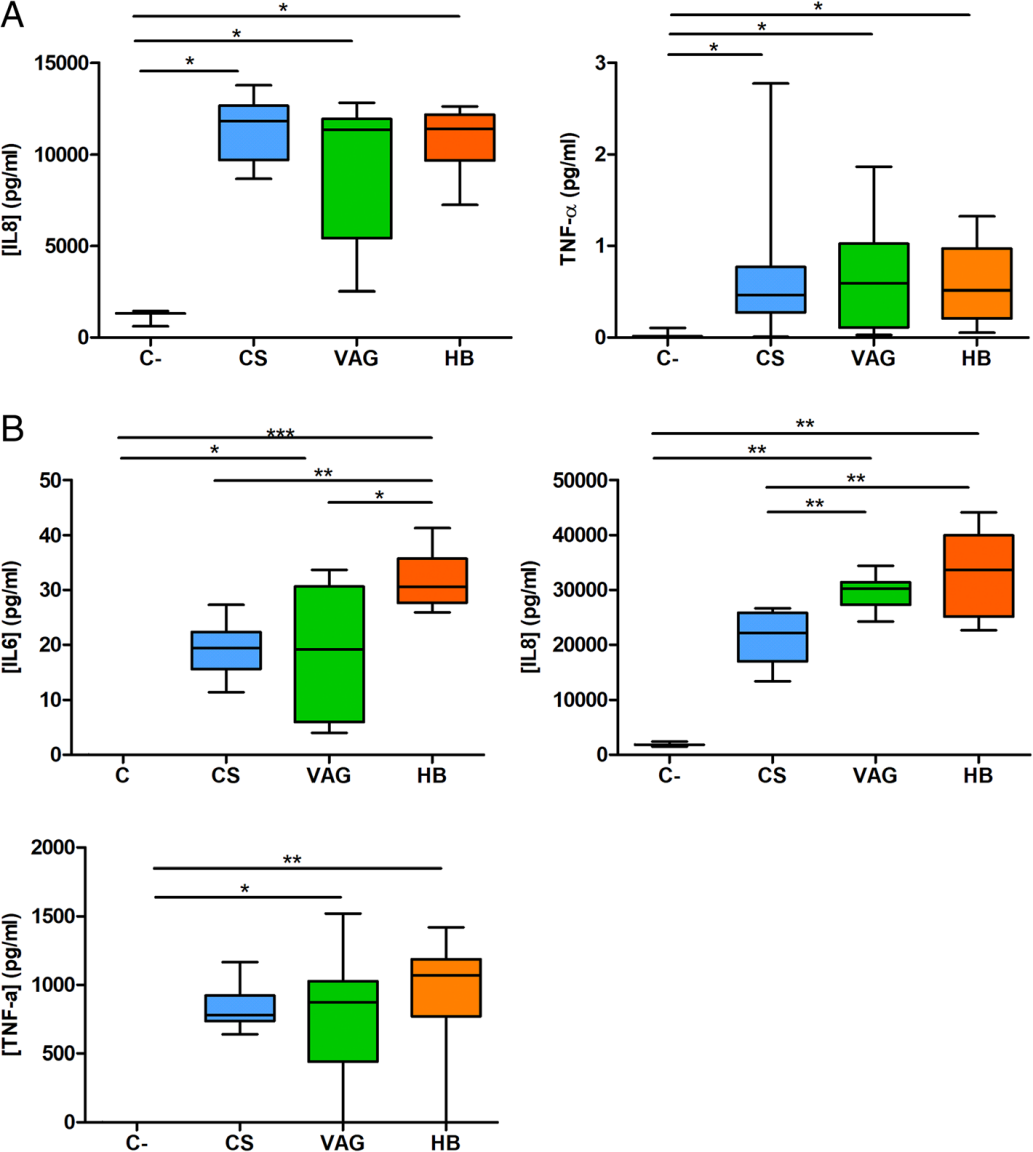
Effect of 1-month infant fecal water exposure in epithelial (**a**) and macrophages-like (**b**) cell lines after 24 h. **a** Cytokine production by HT-29 cells after exposure to fecal water from neonates born by C-Section (CS), vaginal delivery at hospital (VAG), and homebirth (HB). IL6 production in HT-29 cell line was below detection limit. **b** Cytokine production of THP1 cells after 24 h exposure to fecal water of each group. Data was presented as median and whiskers represented the 5–95 percentile. Kruskal-Wallis and Dunn’s post hoc (FDR adjustment) test was used to test the significance of the differences in cytokine response between the groups. *p < 0.*05*, **p < 0.*01*, ***p < 0.*001*. C-section (CS), hospitalized vaginal delivery (VAG), and homebirth (HB)

The HT-29 reporter cells showed increased NF-kB activation independently of the place and mode of delivery in all the groups (data not shown). All fecal supernatants induced IL8 and TNF-α production in the HT-29 cells when compared with control cells exposed to cell culture media (Fig. 5a). No differences were found between the groups in relation to any of the cytokines.

HB fecal supernatants upregulated the *TLR4* mRNA in a 1.405-fold change (*p = 0*.*024*) and the *IRAK4* mRNA in a 2.654-fold change (*p < 0*.*001*) when compared with the control samples. This increase was not observed in the cells exposed to hospital-born (VAG and CS) neonatal samples (Additional file 8). Generally, the fecal supernatants all reduced the mRNA expression of the tight-junction proteins, including zonulin-1 (*HP*) (0.202- and 0.265-fold change in the HB and CS samples, respectively), e-cadherin (*CDH1*) (0.226- and 0.326-fold change in the HB and CS, respectively), and occludin (*OCLN*) (0.192- and 0.253-fold change in the HB and CS). However, no differences were found between the different studied groups.

### HB fecal supernatants trigger higher immune response in macrophage-like cells than CS fecal supernatant

The mode of birth significantly affected the production of cytokines in the PMA-differentiated THP-1 cells (Fig. 5b). The HB and VAG fecal supernatants had a higher pro-inflammatory capacity than the CS samples. Higher levels of IL6 and IL8 were detected after HB (*p = <0*.*001*), followed by the VAG (*p = 0*.*043*) samples, when compared with the CS-born fecal supernatant. The VAG and HB samples triggered a significantly higher response than the control condition for IL6, IL8, and TNF-α, which was not observed in cells exposed to CS samples.

The CS fecal supernatants downregulated the *TLR4* (0.509-fold change, *p = 0*.*006*) and *FOS* mRNA expression (0.238-fold change, *p = 0*.*038*) (Additional file 8). The expression levels were not quantifiable for the interferon gamma (*IFN-γ*) and *IL10* genes in either the HT-29 or THP-1 cell lines following fecal supernatant exposure.

### Triple co-culture system for host–microbiome interaction

#### Place and mode of birth impacts on intestinal barrier function and maturation

**Fig. 6:**
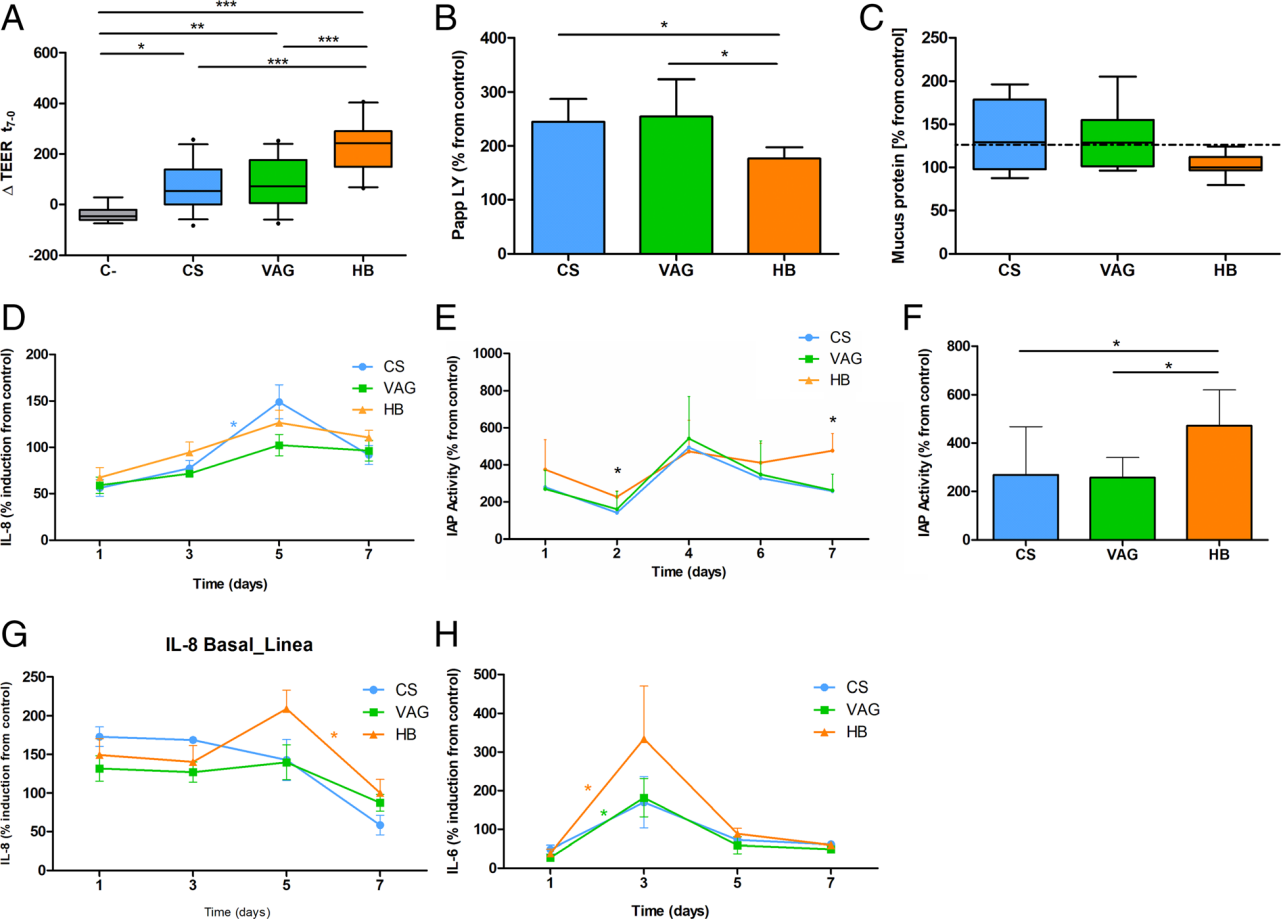
Effect of fecal water long-term exposure (7 days) on the triple co-culture system. **a**, **b** Epithelial barrier function measured as trans-epithelial electric resistance (TEER) (**a**) and Lucifer yellow transport (LY) (**b**). **c** Mucus production by LSTH17 cells after the long-term exposure on the triple co-culture system measured by Bradford assay. **d** Interleukin (IL) 8 production by cells on the apical compartment (CacO-2 and LSTH17) measured by ELISA and expressed as increment respect to control condition. **e**, **f** Intestinal cells functional maturation degree measured as intestinal alkaline phosphatase activity (IAP) on apical compartment during the treatment (**e**) and at final time point (**f**). **g**, **h** Cytokine production in the basal compartment by THP-1 cells. IL-8 (**g**) and IL-6 (**h**) production after fecal supernatant long-term exposure expressed as increment respect to control condition. The treatments were fecal water from infants born by C-section (CS), vaginal delivery at hospital (VAG), and homebirth (HB). Non-normal data was presented as median and whiskers represented the 5–95 percentile while normal data was showed as mean and SD. Kruskal-Wallis/ANOVA and Dunn’s/Tukey’s post hoc (FDR adjustment) test was used to test the significance of the normal/non normal distributed variables between the groups. In the cytokine analysis, the symbol (*) represented variations between time within the same studied group according to the color. *p < 0.*05*, **p < 0.*01*, ***p < 0.*001*

With regard to the integrity of the cell monolayer (Additional file 9), HB promoted a higher increase in the transepithelial electrical resistance (TEER) values after 7 days of exposure (Fig. 6a, b) when compared with hospital birth (VAG [*p < 0*.*001*] and CS [*p < 0*.*001*]). These results were confirmed via the measurement of Lucifer yellow dye (LY) transport through the epithelial layer. Despite all the fecal supernatants triggered an increase in LY transport when compared to control condition (*p < 0*.*05*), hospital-based birth, both VAG (2.1·10^−6^ ± 5.7·10^−7^ cm/s, *p = 0*.*018*) and CS (2·10^−6^ ± 3.5·10^−7^ cm/s, *p = 0*.*033*), led to higher LY transport than HB (1.5·10^−6^ ± 1.7·10^−7^ cm/s).

The intestinal alkaline phosphatase (IAP) activity was also measured in the supernatant of cell cultures from both the apical and basal compartments (Fig. 6e,f). Indicative of functional cell polarization, the IAP activity was significantly higher (*p = 0*.*006*) in the apical compartment at 7 days of treatment, while all the fecal supernatants enhanced the IAP activity when compared with the control. Similar to the TEER and LY results, HB samples induced higher IAP activity than the ones observed in VAG (*p = 0*.*043*) and CS samples (*p = 0*.*049*). Furthermore, higher mucus production was observed in cells exposed to hospital fecal samples (0.92 ± 0.2 mg/ml) when compared with the HB samples (0.69 ± 0.09 mg/ml) (*p = 0*.*006*).

#### Immune system response

The immune response to the fecal supernatants was generally higher in the basal than in the apical compartment. In the simulated intestinal epithelium, the exposure to CS-born fecal supernatants downregulated (0.43-fold change) the *IRAK4* mRNA expression when compared with the control-no stimulus (*p = 0*.*01*) and HB samples (0.45-fold change, *p = 0*.*001*). Contrarily, the CS-born fecal supernatants upregulated the toll-interacting protein (*TOLLIP*) mRNA (*p = 0*.*007*) expression in the HB samples (1.54-fold change) (Table 1).

**Table 1:** Gene expression of cells in the apical and basal compartment in the triple co-culture system after 7 days of exposure

	HB		CS	
	Relative expression	***p*** value	Relative expression	***p*** value
*Apical compartment* (*Caco-2*, *LSTH-17 cells*)				
*HP*	1.139 (0.767–1.627)	*0*.*495*	1.097 (0.957–1.214)	*0*.*106*
*CDH1*	1.054 (0.462–1.675)	*0*.*841*	0.916 (0.736–1.237)	*0*.*399*
*OCLN*	1.163 (0.673–1.534)	*0*.*531*	1.321 (0.867–1.702)	*0*.*083*
*IRAK4* (*#*)	0.94 (0.610–1.168)	*0*.*837*	0.425 (0.273–0.595)	*0*.010*↓
*TLR2*	1.492 (0.837–2.142)	*0*.*181*	1.302 (0.960–1.710)	*0*.*061*
*TLR4*	0.89 (0.722–1.074)	*0*.*201*	0.826 (0.482–1.276)	*0*.*475*
*TOLLIP*	0 (0.000–1.381)	*0*.*509*	1.231 (0.658–2.076)	*0*.*474*
*Basal compartment* (*THP-1 cells*)				
*TLR4*	3.566 (1.103–13.629)	*0*.*094*	1.117 (0.235–7.013)	*0*.*896*
*TLR3*	1.346 (0.939–1.853)	*0*.*378*	3.546 (1.712–8.099)	*0*.*094*
*TOLLIP*	5.182 (3.667–9.351)	*< 0*.001*↑	7.186 (4.388–11.727)	*0*.010*↑
*IL10*	7.888 (3.496–31.769)	*< 0*.001*↑	1.737 (0.912–5.478)	*0*.*061*

Total RNA was extracted from cells treated with homebirth (HB) samples and C-section (CS) fecal supernatant. For each condition, *n* = 6 in triplicates, data was presented as fold change expression (95% C.I). Values show relative expression of each condition compared to control. *P < 0*.*05* (*) and blond letters marked significant differences between treatment and control, up (↑) or down (↓) regulation was represented by the arrows. Symbol # represented genes expression that was different between HB and CS

IL8 was detectable at all time points in the apical compartment (Fig. 6d). Generally, the IL8 exhibited a gradual increase until the fifth day of exposure (126 ± 39% average increase for the three groups). Thereafter, the IL8 levels decreased until the end of treatment (7 days, 99 ± 24% average of three groups). The IL6 concentration was only above the detection limit at the final time point (7 days), and there were no differences between the groups.

In the basal compartment, similar to the epithelium-like layer, we observed a 7.2-fold change in the downregulation of the *TOLLIP* mRNA expression after exposure to CS-born samples (*p < 0*.*001*) (Table 1).

IL8 release was observed at 24 h (151.3 ± 42.7% increase from the control), with the maximum being seen after 5 days of treatment (164 ± 65% increase). At day 7, the IL8 levels reached similar values to those seen on the first day of treatment (82 ± 67% increase) (Fig. 6f–j).

The IL6 production by the THP-1 cells showed a late response to fecal supernatant exposure, lasting from day one of treatment until day three (218 ± 223% increase from control); however, the increase was only significant for the HB (*p = 0*.*003*) and VAG (*p = 0*.*017*) samples. At the subsequent time points, the IL6 release was stabilized to concentrations similar to those seen on day one (61.45 ± 11.69% for CS, 48.88 ± 11.14% and 59.97 ± 13.4% for HB). No significant differences were observed between the groups in terms of the cytokine production patterns over the 7 days.

## Discussion

In this study, we evaluated gut microbiota evolution during the first month of life in CS-delivered and both HB and hospital-based VAG infants. Our results highlight the relevance of perinatal factors to the gut microbial colonization pattern, which affects the innate defensive mechanisms and functional maturation at the intestinal level.

CS procedures are associated with specific conditions, including the use of antibiotics, longer hospitalization, low neonatal–maternal contact, and the delayed initiation of breastfeeding [35, 36], which may influence microbial colonization patterns [26]. A higher percentage of bottle or mixed feeding is commonly observed in CS-born infants [35], including the studied cohort. In our cohort, the HB infants showed a higher rate of exclusive breastfeeding than the hospital-born infants (95% in HB versus 67% and 61% in CS and VAG at hospital, respectively). Furthermore, the practices associated with hospital delivery, such as antibiotic use, vaginal cleansing, controlled maternal food intake and mobility, oxytocin administration, or anesthesia, may affect the maternal microbiota and, consequently, mother–infant microbial transmission. Those factors together with prematurity would have a relevant impact on infant development that remains poorly understood. Additionally, these birth-related factors may affect the microbial establishment process and cannot be independently studied. Therefore, our results sum up the consequences of all the concomitant factors affecting the different groups of study.

The hospital environment, especially in the case of CSs, can be considered highly intervened condition characterized by the high pressure of antibacterial therapy and instrumentalization. HB rates have increased in Europe over recent decades, ranging from 0.1% in Sweden to almost 20% in the Netherlands [37]. However, limited information concerning the possible benefits of HB is currently available due to the lack of adequate randomized clinical trials meaning that it is not possible to determine the risk of neonatal–maternal mortality and morbidity during HB [38]. Further, little is known about the impact of a non-hospital environment on infant gut colonization.

We observed that the mode and place (hospital versus home) of birth shaped the neonatal microbiota. The microbiota of hospital-delivered infants was enriched with gram-positive anaerobic cocci, including the *Peptoniphilus* and *Finegoldia* genera, while those of HB infants exhibit higher relative abundances of species from the *Enterococcus* and *Bifidobacterium* genera.

In all three groups, time was the main factor affecting the composition of the infant microbiota, with a significantly different pattern between groups observed at birth, but not at the subsequent time points. Primary events concerning gut microbial colonization may impact the assembly and acquisition of the early microbiome, likely having long-lasting consequences for gut ecology [29].

At delivery, we observed higher diversity indices in the neonatal microbiota than at the subsequent time points; however, the quantitative total bacteria count was lower at delivery. This is likely caused by bias in the sequencing data obtained from low biomass samples and by higher contact with environmental contaminants. The HB infants had lower microbial diversity but a higher total number of bacteria than the hospital-born infants, likely due to the latter having increased contact with bacterial environmental contaminants [6].

We found clearly identifiable differences in the global microbiota structure during time between the three groups, even at the phylum level, which is in agreement with previous studies [4, 6, 34]. Interestingly, the differences in the colonization patterns become more pronounced over time, at least during the first month of life, indicating the importance of birth-related events to neonatal colonization processes, which may affect the microbiome composition in later developmental stages. Altered microbial colonization has been also observed in preterm infants compared to term infants, and these microbial shifts have been mainly linked to antibiotics exposure, cesarean section, hospitalization, and use of formula feeding. Regarding term infants, some studies have shown shifts related to delivery mode in term infant microbiota up to 3 months post-delivery [8, 39]; other authors observed no differences in terms of microbiota composition in neonates born by CS beyond the first days of life [40]. Thus, the duration of the shifts in infant microbiota associated to delivery mode has reported contradictory results and needs to be further evaluated.

Similar to prior studies, we found that CS-born infants showed higher relative abundances of *Clostridium* and *Klebsiella* and lower species from the *Bifidobacterium* genus [6]. We observed that both VAG and HB infants had higher number of *Bifidobacterium* species than CS-born neonates. The depletion of the *Bifidobacterium* species in the gut environment has been associated to immune-related diseases [41, 42], and members of this genus are commonly used as probiotics due to their capacity to modulate microbiota–immune system homeostasis [43].

### Could these shifts in the microbiota composition alter the functional profile of the neonatal microbiota?

Descriptive and observational studies are relevant to understanding microbiome evolution during the neonatal period; however, mechanistic studies of the host–microbiome interplay during early life are still required. Thus, microbiota functional analysis has been proposed as a tool for clarifying the host–microbiome interactions [44]. Our results of the predicted metagenome from 16S rRNA sequencing data showed functional differences between the place and mode of birth in the infant microbiota at delivery, 7, and 31 days. Several amino acid (AA) biosynthesis routes were over-represented in the microbiota from vaginal-delivered infants when compared with the CS-born infants, including tryptophan-related paths. It is known that AAs serve as regulators of several metabolic pathways in the host [45] and more specifically tryptophan interacts with the immune system through microbial serotonin production [46], regulation of TLRs, or interacting with the aryl hydrocarbon receptor–microbiota–immune system path [47, 48].

We also found that vaginal-delivered infants, especially HB babies, had a microbiota enriched with LPS biosynthesis-related functions. Similarly, Wampach et al. found differences in the earliest functional profile according to the delivery mode, including LPS biosynthesis routes being enriched in vaginal deliveries when compared with CS-born neonates [49], which may influence immune system maturation and neonatal health. Despite these observations, these results need to be further evaluated since the prediction of microbiota functionality has been shown to report results that may be not conclusive [50].

### Do these differences in the microbiota influence the host immune system response?

Despite studies in animal models highlighting the possible effects of microbial colonization patterns on the host gut epithelium maturation and immune system response, little evidence is available from human studies. To the best of our knowledge, this is among the first studies to address this important issue. The intestinal epithelium is the gateway through which gut microbiome–host crosstalk effects intestinal functionality in the form of enterocytes maturation, mucus production, or epithelial barrier development, being a key anatomical location for host–microbiome interplay.

The samples from HB infants exhibited a higher immune stimulatory capacity than those from hospital-born infants (both VAG and CS), with an increased ability to induce the expression of immune system-related genes (*TLR4* and *IRAK* mRNA) and cytokine responses in the HT-29 and THP-1 models, including IL6 and IL8. In concordance with our results, Wampach et al. identified the higher immunostimulatory potential of the microbiota of vaginal-delivered infants when compared with CS-born infants, although they used LPS purified from infant fecal samples and primary human macrophages differentiated into dendritic cells [49]. Combellick et al. noted the higher expression of *TLR4* and *IL8* mRNA by the HT-29 cell line following exposure to sterile fecal supernatant from HB infants when compared with hospital-born infants. However, they also found mRNA upregulation of the anti-inflammatory cytokine *TGF-β* in hospital-born neonates [34]. We observed that the THP-1 cell line response was more affected by the mode and place of delivery than the HT-29 cell line, which highlighted the importance of the epithelial integrity and the innate immune system on the in vitro assessment of the host-microbiome interplay.

Most prior studies with similar objectives involved acute exposure on unique cell lines. We hypothesized that acute exposure to microbial products could not accurately reflect the biological effect of microbial metabolites and so proposed long-term (7 days) in vitro exposure assays, including the crosstalk between different cell types, which enabled us to obtain personalized results for each participant, thereby translating the individual signatures to the in vitro system.

In our model, the HB fecal supernatants induced higher gut barrier integrity (Fig. 6) and functionality (IAP; Fig. 6) following a time-dependent response that highlighted the relevance of the dynamics of host–microbiome interplay. Interestingly, the impaired closure of gut mucosal membranes has been shown, alongside higher intestinal permeability, in preterm infants who received formula feeding rather than breastfeeding [51]. This increased permeability could be related to allergic diseases in non-breastfed children [52] and other health disorders [53]. In addition, the HB samples induced the expression of anti-inflammatory molecules (e.g., *IL10*, *TOLLIP*) to a higher extent than the CS samples, indicating negative feedback on the inflammatory signaling in the gut. Specifically, IL10 downregulate the microbiota-activated mucosal inflammatory cytokines, reinforce the gut epithelium barrier, and control gut permeability, all essential factors to maintaining intestinal homeostasis [54]. Another key element of the innate immune response of the gut is the protective mucus layer covering the epithelium [55]. Higher mucus production was observed after cell-exposure to fecal supernatants obtained from hospital-born infants when compared with HB infants. Both microbiota [56] and TLR expression [57] are involved in the regulation of mucus production. Despite lower mucus production in the gut being associated with disease phenotypes (e.g., inflammatory bowel disease, higher susceptibility to bacterial infections) [58] in adults, little is known about the role of mucins in neonatal colonization processes. We hypothesize that a penetrable mucus layer in newborns would allow for microbial colonization and interaction with the epithelium during the immune-priming window.

Many researchers have discussed the possible relationship between CS and altered immune system development [26]. Generally, CS delivery is associated with the poor stimulation of the immune system [59–61]. Some researchers have proposed non-diverse environments in early life, including delivery, to play a role in immune maturation and triggering. Furthermore, recent evidence has shown that early exposure to rural areas or farm environments could affect microbial composition and diversity [62], which may be linked to a reduced risk of suffering atopies in adulthood [63]. Kirjavainen et al. recently described how a farm-like indoor microbiota could also decrease the asthma risk in a non-farm environment [64].

However, most of these results were derived from observational studies, as very few mechanistic analyses have been conducted to date. Our results suggest a possible link between CS and the delayed maturation of intestinal function and the innate immune system. Such a link could play a significant role in the diseases associated with intervention-based deliveries, including autoimmunity, allergy and other immune- and metabolic-related disorders.

In this regard, CS-born infants from our cohort showed higher BMI and W/L *z*-scores during the first 18 months of life. Other researchers have reported similar results, associating CS with the risk of overweight in children [65, 66]. These results could indicate the relevance of priority events in infant health, including those altering microbial colonization, thereby supporting the early programming hypothesis [67, 68]. Researchers have described how antibiotic therapy during early life modulates weight gain in different ways depending on the antibiotic dose in both animal [69] and human epidemiological studies [66]. It has been suggested that high-dose antibiotics can cause important reductions in the microbiota population, which may be related to the weight loss observed in some studies [70]. However, lower antibiotic doses would cause microbiota composition shifts, more than population size variation, and trigger the weight gain shown in the above-mentioned studies. Thus, it remains to be discovered whether the proposed mechanism could be extended to other perinatal factors that also disrupt the microbiota composition and transmission.

The limitations of this study include the low number of participants and the possible confounding factors not included in the analysis (e.g., maternal diet, lifestyle or number of siblings, pets among others). Our microbiota analysis was based on the taxonomic profile obtained via 16S rRNA gene sequencing, which offers less resolution than complete shotgun metagenome sequencing. As we used sterile fecal supernatant, we observed the effects of soluble bacterial metabolites and also, of non-bacteria-related products, including growth factors or eukaryotic extracellular vesicles, which may have influenced the observed results. The use of cell lines may hamper the translational results, although it offers a reproducible and economically viable strategy for further testing on more physiologically relevant models. Among the strengths of the study are the inclusion of three groups and the comparison of CS and vaginal delivery at both hospital and home, including 18 months of follow-up. We performed the cellular exposure assays in cellular models with different degrees of complexity and different exposure times, including the epithelial barrier function and maturation as relevant targets, together with the innate immune response. Yet, fecal supernatants contain a complex array of molecules representative of the in vivo condition, which retain inter-individual differences and features.

## Conclusion

Our results may provide a mechanistic linkage between studies associating CS and immune-related diseases with colonization pattern alterations, although we cannot rule out other possible factors that might participate in the process. The study has shed light on the effects of hospitalization and HB on neonatal microbial colonization and on the possible effect on innate immune system development, specifically at the intestinal level. The results highlight both the importance of host–microbial contact during the first month of life and the dynamism of the process. However, further research is needed to determine the impact of these observations in neonatal in vivo clinical conditions. Such knowledge would facilitate the design of strategies for adjusting medical practices with the aim of reducing intervention during the birth process and ensuring the correct initiation of bacterial colonization and, consequently, the immune system response during early life.

## Methods

### Subjects and sampling

A prospective cohort study was conducted to compare the intestinal microbiota of delivered infants born at the hospital (VAG, *n* = 92 and CS, *n* = 65) and at home (HB, *n* = 24). Infants with available biological samples at birth, 7 days, and 1 month, together with clinical data, were included.

Ethical approval for the study was obtained from the Ethics/Bioethics Committee for Clinical Research of Hospital La Fe, Hospital Clinic, Parc de Salut MAR, and CSIC (Consejo Superior de Investigaciones Científicas) [ClinicalTrial.gov NCT03552939].

Parents gave written informed consent before enrollment. Methods were performed in accordance with the relevant guidelines and regulations published previously [71]. Women had healthy pregnancies with non-declared pathology and neonates were delivered at term without complications during labor or CS intervention. All the C-section mothers received intravenous intrapartum antibiotics during the intervention. In the case of vaginal deliveries that required antibiotherapy, it was administrated during the dilatation process.

Neonatal fecal samples were obtained within the first 24 h after delivery and stored in sterile conditions. A sterile, cotton-tipped swab was used for sampling neonatal fecal samples by trained clinical personnel at delivery room as detailed previously [71]. Subsequently, fecal samples were collected in sterile containers by the parents at home using detailed instructions at 1 week and 1 month after birth. Samples were immediately stored at − 20 °C, transported within 24 h after collection, and stored at − 80 °C until analysis.

Pregnancy, intrapartum variables, and anthropometric data were recorded (Additional file 1). Maternal age, maternal pre-pregnancy weight, weight gain over the pregnancy, maternal smoking status, mode of delivery, place of birth (home or hospital), birth weight and length, sex of the neonate, birth instrumentalization, maternal antibiotic exposure during pregnancy, and maternal/infant antibiotic use at birth were also collected.

Infant length and weight were also registered at birth, 1, 6, 12, and 18 months. *Z*-scores of anthropometric measures were electronically computed using WHO Anthro software (www.who.int/childgrowth/software/en/). The WHO Child Growth Standards provide child growth measures standardized by age and sex using *z*-score.

### Fecal DNA extraction

Total DNA was extracted from the fecal material (approx. 50–100 mg) using the Master-Pure DNA extraction Kit (Epicentre, Madison, WI, USA) following the manufacturer’s instructions with the following modifications: samples were treated with lysozyme (20 mg/mL) and mutanolysin (5 U/mL) for 60 min at 37 °C and a preliminary step of cell disruption with 3-μm diameter glass beads during 1 min at 6 m/s by a bead beater FastPrep 24-5G Homogenizer (MP Biomedicals). Purification of the DNA was performed using DNA Purification Kit (Macherey-Nagel, Duren, Germany) according to manufacturer’s instructions. DNA concentration was measured using Qubit® 2.0 Fluorometer (Life Technology, Carlsbad, CA, USA) for further analysis.

### Sequencing and bioinformatics analysis

DNA libraries were performed with the amplification of the V3-V4 variable region of the 16S rRNA gene following the 16S rDNA gene Metagenomic Sequencing Library Preparation Illumina protocol (Cod. 15044223 Rev. A). The primers were selected from [72]. A multiplexing step was conducted by the NextEra XT Index Kit (FC-131-2001) (Illumina, San Diego, CA, USA) and DNA quality of the library PCR product was measured by a Bioanalyzer DNA 1000 chip (Agilent Technologies, Santa Clara, CA, USA) to verify the size; the expected size on a Bioanalyzer trace is ~ 550 bp. The libraries were sequenced using was a 2 × 300 bp paired-end run (MiSeq Reagent kit v3) on a MiSeq-Illumina platform (FISABIO sequencing service, Valencia, Spain) according to manufacturer instructions. Obtained reads were searched for residual adaptors using the program Trimmomatic v. 039 [73].

Quality-trimmed and filtering was assessed using DADA2 v. 1.12.1 pipeline [74]. After quality examination, reads were trimmed at the 270th and 210th nucleotide in forward and reverse position, respectively. Additionally, adapters were also removed in the filtering process and a maximum of 2 expected errors was considered. The following denoising and merging steps were performed, and chimeras were also removed. Taxonomic assignment was conducted using the Silva v132 database with the addition of the specie level classification by the same database.

Taxa occurring < 3 reads in at least 10% of the total samples number and those representing less than 0.01% of the reads across all the samples were filtered. Furthermore, the *decontam* package v. 1.4.0 [75] in R environment [76, 77] was used to determine the presence of potential contaminants-related sequence. Samples with less than 1000 reads were also removed from the final analysis (*n* = 6). One of those samples was from a vaginally born infant with samples only at delivery time and this infant data was eliminated in further analysis. The final ASV (amplicon sequence variance) table is listed in Additional file 11. The 16S rRNA gene sequence data generated is available through NCBI Sequence Read Archive Database under project accession number BioProject ID PRJNA614975.

Predictive inferred functional analysis was performed using PICRUST v. 1.1.4 pipeline [78] and the linear discriminant analysis effect sized (LEfSe) analysis was performed for the biomarker discovery using a size-effect cut-off of 3.0 on the logarithmic LDA score [79] using the R code available in the yingtools2 package (https://rdrr.io/github/ying14/yingtools2/man/lefse.html) and the Dr. Huttenhower’s lab galaxy repository of bioinformatic tools.

### Bacterial quantification by quantitative PCR analysis

A small subset of samples (*n* = 248) according to DNA availability (delivery *n* = 78; 7d *n* = 100; and 31d *n* = 86) were used for the specific bacterial count determination by the qPCR. Total bacterial and *Bifidobacterium* genus counts were measured by quantitative system based on the amplification of specific 16S rRNA gene region by use of Light Cycler 480 Real-Time PCR System (Roche, Basilea, Switzerland). The (details in Additional file 11) reaction mixture consisted SYBR Green I master mix (Roche, Basilea, Switzerland), 0.25 μM of each specific primer set, and 1 μl of DNA. The amplification process consists of one cycle at 95 °C for 5 min, followed by 40 cycles at 95 °C for 20 s, annealing temperature (Additional file 12) for 10 s, and 72 °C for 10 s. Melting curves were also assessed to test the specificity of the reaction. Standard curves for the specific targeted bacterial group were generated using *Ct* values and the calculated gene copies numbers were determined based on the fragment amplification length.

### Cell culture

All the reagents for cell culture were purchased from Sigma-Aldrich, Spain, otherwise stated.

#### NF-κB-SEAP HT29 reporter cells

The HT-29-transfected cell line was previously established in the Laboratory of Lactic Acid Bacteria and Probiotics of IATA-CSIC, by stable transfection of HT-29 cells with a NF-κB- secreted alkaline phosphatase (SEAP) plasmid (pNiFty2-SEAP; Invitrogen, Carlsbad, CA, USA) as a reporter. Cell maintenance was performed in 75 cm^2^ flask with Dulbecco’s Modified Eagle Medium (DMEM) High Glucose medium supplemented with 1% (v/v) L-Glutamine 200 mM, 1% (v/v) Na-Pyruvate, 1% (v/v) penicillin/streptomycin, and 10% (v/v) of inactivated Fetal Bovine Serum (Biowest). Zeocin (200 μg/ml) (InvivoGen) was added to the medium for the clone selection in each passage. All cultures were used between the passage 15 and 20.

#### THP-1 cells

The THP-1 cells were obtained from the European Collection of Authenticated Cell Cultures (THP-1 ECACC 88081201, Public Health England, UK). Cell maintenance was carried out as described in Boudish et al. [80]. All cultures were used between the passage 40 and 50.

#### Caco-2 and LS17T cells

The Caco-2 (ECACC 86010202, Public Health England, UK) and LS174T (ECACC 87060401, Public Health England, UK) cells were obtained from the European Collection of Authenticated Cell Cultures. Caco-2 cells were maintained as described in [81]. LS174T cells were maintained in 25 cm^2^ flasks with Eagle’s Minimum Essential Medium (EMEM), supplemented with 1% (v/v) GlutaMax, 1% (v/v) Non-Essential Amino Acids (NEAA), 10% inactivated Fetal Bovine Serum (FBS), and 1% (v/v) penicillin/streptomycin. Medium was refreshed every 2 days, and cells were subcultured when they reached 80% confluence, as described in [81]. All cultures were used between the passage 40 and 50. Cell morphology was analyzed and checked by phase- contrast microscopy (Olympus CKX41, Olympus Corporation, Tokyo, Japan).

### Stimulation of HT-29 and THP-1 cell line

To investigate the role of microbial shifts on NF-κB activation and innate immune response, NF-κB-HT-29-reporter cells and macrophage-like cell line (THP-1) were exposed to filtered fecal supernatant obtained from a subset of samples of each group (*n* = 4, total *n* = 12) at 1 month. Samples were randomly selected among the samples with fecal material availability.

Fecal supernatants prepared as described above (*n* = 4 individuals of each group, total *n* = 12) were filter-sterilized (0.22 μm PES; Sarstedt SA, Barcelona, Spain) and exposed to the cells as described below. pH adjustment to 7.0–7.2 was performed when required with 0.5 M filter-sterilized NaOH (Panreac, Barcelona, Spain) and buffered with HEPES (1% v/v).

HT-29 cells were seeded at 7 × 10^4^ cells/well in 96-wells plates and incubated for 24 h in DMEM High Glucose medium without FBS supplementation. Cells were exposed to filtered fecal supernatant obtained from the three studied groups diluted in 1:10 v/v in DMEM with FBS. Supernatants were collected after 24 h of stimulation and SEAP activity was measured using p-nitrophenyl phosphate according to manufacturer’s instructions (Thermo Fisher Scientist, Waltham, USA). The signal was quantified using a Spectrostar Nano microplate reader (BMG Labtech, Ortenberg, Germany) at 405 nm.

THP-1 cells were seeded at 5 × 10^4^ in 96 wells plates in RPMI 1640, supplemented with 100 ng/mL of phorbol 12-myristate 13-acetate (PMA). After 48 h, cells were refreshed with RPMI without PMA and incubated for 4–5 days, to allow macrophage-like differentiation of THP-1 cells. Thereafter, the cells were exposed fecal supernatants diluted 1:20 (v/v) in RPMI and incubated for 5 h. Next, cell culture supernatants were collected, and cells were washed twice with PBS. Cells and supernatants were stored at − 80 °C for gene expression determination and cytokine quantification, respectively.

### Triple co-culture in Transwell plates: long-term stimulation

A subset of samples from each group at 1 month of life (*n* per group = 3, total *n* = 9) were selected to investigate in a more physiologically model the long-term effect of the differences observed in microbiota composition of the infants on the gut barrier and innate immunological state.

Cell differentiation and the posterior tests were carried out in double chamber wells (Corning® Transwell®-6 well, pore size 0.4 μm; Costar, NY) equipped with separate apical and basolateral compartments and a porous support on which the Caco-2 and LS174T cells grow into a monolayer. The cells were seeded at a density of 7.5 × 10^4^ cells/cm^2^, in a proportion of 90/10 Caco-2/LS174T in supplemented DMEM. After 24 h, apical media was refreshed, basal media was removed, and THP-1 cells were seeded in the bottom of the 6 well plate at a density of 1·10^5^ cells/cm^2^ in RPMI 1640 media containing 100 ng/mL of phorbol 12-myristate 13-acetate (PMA).

After 48 h of incubation, both apical and basolateral compartments were refreshed with DMEM or RPMI 1640 without PMA and without antibiotics, respectively. Thereafter, the triple co-culture was maintained for 4 days more and refreshments of the apical and basal media were done every 2 days.

Filter-sterilized fecal supernatants selected from each studied group (*n* = 3 individuals, total *n* = 9) were diluted 1:20 in cell culture media without antibiotic/antifungals and added to the apical compartment of the triple co-culture model (1.5 mL). Treatments were maintained for 7 days, with daily refreshments of the apical and basolateral compartments, with fecal supernatants or RPMI 1640, respectively. Aliquots of the culture supernatant from apical and basolateral media were stored at − 80 °C for cytokine measurements.

### Epithelial barrier function: trans-epithelial electrical resistance (TEER) and apparent permeability

The monolayer integrity was assessed by measuring the trans-epithelial electrical resistance (TEER) and the apparent permeability (Papp) of the paracellular transport marker Lucifer yellow (LY).

A Millicel-ERS (Millipore Corporation, Spain) was used for the TEER measurements. Measurements of the TEER were performed every day at the beginning of the exposure to sterilized fecal supernatant (7 days post-seeding) and every day until the end of the assay (15 days post-seeding). TEER values are reported as delta (Δ) of the initial time point before of the exposure and final time point (7 days). TEER values (ohms/cm^2^) are presented in Additional file 9.

Papp of LY was measured by adding the marker (100 μM) to the apical compartment of the wells. After 30, 60, and 120 min, 100 μL of medium was removed from the basolateral compartment and replaced with an equal volume of fresh medium (supplemented RPMI-1460 without antibiotics). LY fluorescence was measured at an excitation/emission wavelength of 485/520 nm in 96 black plates (Greiner), using a microplate fluorescence reader CLARIOstar Plus (BMG Labtech, Ortenberg, Germany). A calibration curve (0, 5, 10, 25, 50, and 100 μM) for LY quantification was run in duplicate in each reading. The Papp coefficients were calculated as previously described in [82].

### Mucus production and intestinal alkaline phosphatase (IAP) determination

At day 15 post-seeding (7 days of fecal supernatant exposure), the Transwell inserts containing Caco-2/LS174T cells were incubated with 1 mL of 10 mM of N-acetyl cysteine (Sigma) for 1 h at 37 °C, 95% humidity in DMEM, adjusted at pH 7–7.2, and the mucus produced by the cells was collected recovering the media and washing once with 0.5 mL of DMEM. The solution containing mucus was concentrated using a Vacuum Concentrator (Eppendorf, Hamburg, Germany) until dryness and re-suspended overnight in 100 μL of PBS at 4 °C. The amount of mucus was measured by a Bradford protein quantification assay, following the manufacturer instructions. Blanks containing cell culture media were subtracted and a standard curve of bovine serum albumin (BSA) was used for the calibration curve (0–5 mg/ml).

Intestinal alkaline phosphatase (IAP) activity was assessed in the apical and basal compartment supernatants by enzymatic assay following manufacturer’s instructions (Sigma-Aldrich, Missouri, USA) scaling the reaction to 100 μl using 4 μl of cell supernatant. Results were read in a SpectroStar Nano (BMG Labtech, Ortenberg, Germany) at 405 nm.

### Cytokine quantification in cell supernatant

IL6, IL8, and TNF-α released by the cells after acute and long-term exposures to fecal supernatants were quantified by Enzyme-Linked ImmunoSorbent Assay (ELISA), following manufacturer instructions. Human IL8, TNF-α, or IL6 Uncoated ELISA kit (Invitrogen, Carlsbad, CA, USA) were used for the cytokine determination. In the long-term exposure, cytokine released values were expressed as percentage of variation comparing each treatment to control in order to avoid time-dependent effect. Samples were diluted in assay buffer to adjust the concentration to the linear range of the standard curve.

### Gene expression by real-time RT-qPCR

HT-29 and THP-1 cells from the acute exposure as well as those from tripe co-culture system were collected by scraping at the end of the treatment. RNeasy mini kit (Qiagen, Hilden, Germany) was used to RNA extraction following the manufacturer’s instructions. Total RNA was converted to cDNA by Transcriptor First Strand cDNA Synthesis Kit (Roche, Basilea Switzerland) adjusting to the same amount of RNA for each cell type. RT-qPCR analysis was performed using 1 μl of resulted cDNA reaction and 0.25 μM of the specific primers using Lightcycler 480 SYBR Green I master mix (Roche, Basilea, Switzerland). Plates were read in the LightCycler 480 (Roche, Basilea, Switzerland) at annealing temperature of 58 °C. Sequences of the primers used in the study are listed in Additional file 12. Genes related with microbes sensing as TLRs and related transcription factors were analyzed and Actin (ACTB) gene was used as housekeeping gene expression, except for the studies in THP1 cell line in the triple co-culture system where hypoxanthine phosphoribosyl transferase (HPRT) gene was used as reference gene for showing higher stability between samples than Actin gene.

### Statistical analysis

Chi-squared test was used to assess differences in the categorical variables of the studied population and ANOVA or Kruskal-Wallis, followed by a Dunn’s post hoc test, was used for the continuous variables according to the distribution of the data. Normality distribution was tested by Shapiro-Wilk test.

Sequencing data were transformed to relative abundance before further analysis. Pyloseq v. 1.28.0 [83] and vegan v. 2.5-6 [84] packages were used for the analysis conducted in the sequencing data, including alpha diversity estimation. For alpha diversity analysis, samples were rarefied with a 90% of the minimum sample depth. Kruskal-Wallis followed by Dunn’s post hoc tests were performed to detect significant differences in the gut microbial alpha diversity according to categorical variables studies using vegan v. 2.5-6 and PMCMRplus v. 1.4.4 packages. Spearman correlations were used to find associations between alpha diversity measures and continuous variables of the population, including gestational age or maternal body mass index, among other, and also between microbial species.

For beta diversity analysis, permutational multivariate analysis of variance (PERMANOVA) was conducted to assess the effect of the studied factors on the neonatal gut microbial composition at ASV level (Bray-Curtis distance) and its functionality. For each factor, differences in the dispersion were also tested. In groups with different dispersions, ANOSIM test was also addressed. Calypso web platform v. 8.56 [85] was used for visualizing the multivariate analysis. The clustering of the samples according to the different studied variables, including mode and place of delivery was performed by discriminant analysis of the principal components (DAPC) at ASV level. Calypso platform was also used for Venn diagrams plotting.

Kruskal-Wallis test followed by a Dunn’s post hoc test with the FDR method for multiple comparisons correction was applied to find significant differences in gut microbial composition or functionality (KEGG categories) between studied groups at each time point.

For RT-qPCR analysis, LC480 Conversion version 2014.1 and LinRegPCR v. 11.0 software [86, 87] were used for efficiency calculation and gene expression data were analyzed by REST2009 [88]. Statistical analysis of data from in vitro experiments and triple co-culture system was performed by GraphPad software v. 5.04 (GraphPad Software, La Jolla CA, USA). Unpaired *t* test was used for statistical analysis of mucus production, IAP, TEER, and apparent permeability. Kruskal-Wallis and Mann-Whitney test was used for ELISA measurements, considered as non-parametric data. A *p < 0*.*05* was considered as a threshold to accept a statistically significant difference. All multiple comparisons were adjusted by false discovery rate (FDR) adjustment method. Each cell culture experiment was performed in triplicate. The R code used in this analysis is available in Additional file 13.

## Supplementary Information

**Additional file 1.** Characteristics of studied population according to place and mode of delivery.

**Additional file 2.** Neonatal fecal microbiota diversity and richness of meconium and infant fecal samples at 7 and 31 days.

**Additional file 3.** Relative abundance of neonatal fecal microbiota along the first month of life

**Additional file 4.** Taxonomic biomarkers of microbiota composition of each group depending on place and mode of delivery.

**Additional file 5.** Core group of neonatal microbiota composition at genus level over the first moth of life.

**Additional file 6.** Quantitative analysis of intestinal microbiota from infants born at hospital (vaginal and C-section delivery) and at home across the first month of life.

**Additional file 7.** Microbial functions related to amino acids metabolism computationally predicted present in neonatal microbiota along the first month of life.

**Additional file 8.** Gene Expression of HT-29 and THP-1 cells after 24 h of fecal supernatant exposure.

**Additional file 9.** Epithelial barrier function and maturation of simulated intestinal epithelium of the triple co-culture during the long-term exposure

**Additional file 10.** Flow chart of study participants.

**Additional file 11.** Amplicon sequence variant (ASV) table.

**Additional file 12.** Primers of 16 s rRNA gene of prokaryotic targets and human genes tested by qPCR.

**Additional file 13.** R code used in the study and links of consulted GitHub repositories (R 5 kb)

## Data Availability

The dataset supporting the conclusions of this article is available in the NCBI’s Sequence Read Archive (SRA) repository, BioProject ID PRJNA614975 (http://www.ncbi.nlm.nih.gov/bioproject/614975). We acknowledge support of the publication fee by the CSIC Open Access Publication Support Initiative through its Unit of Information Resources for Research (URICI).

## Abbreviations

C-section: Cesarean section
VAG: Vaginally birth
HB: Home birth
BMI: Body mass index
W/L: Weight for length
TLR4: Toll-like receptor 4
TEER: Trans-epithelial electrical resistance
Papp: Apparent permeability
LY: Lucifer yellow
IL: Interleukin
