# Supplementary material for: Perinatal environment shapes microbiota colonization and infant growth: impact on host response and intestinal function
Source: Microbiome. 2020 Nov 23;8:167. doi: 10.1186/s40168-020-00940-8 (PMC7685601; doi:10.1186/s40168-020-00940-8)
Supplement: Supplementary file 2 — Additional file 1. Characteristics of studied population according to place and mode of delivery. [file 40168_2020_940_MOESM1_ESM.pdf]

Additional file 1. Characteristics of studied population according to place and mode of delivery.

		C-section (n=65)	Hospital Vaginal birth (n=92)	Home birth (n=23)	p-value
**MATERNAL DATA**					
**Maternal age (years)**		35.5 [33-38]	34 [31-37]	34 [32-36]	.056
**Pre-gestational BMI (Kg/cm^2)**		23.19 [21.5-25.9][a]	21.69 [20.37-23.85][b]	22.77 [19.75-24.77][ab]	**.027***
**Weight gain over pregnancy (Kg)**		13.42 ± 4.91	12.16 ± 4.01	13 ± 4.33	.315
**Antibiotics during pregnancy**		27 (41.5%)[a]	31 (34%)[a]	1 (4.34%)[b]	**<.001***
**Antibiotics during delivery**		65 (100%)[a]	10 (10.9%)[b]	0 (0%)[c]	**<.001***
**NEONATAL DATA**					
Delivery	Gestational age (weeks)	39 [38-40][a]	40 [39-40][a]	40 [39-41][b]	**.004***
	Neonatal weight (g)	3305 [2925-3710]	3200 [2965-3440]	3360 [3145-3630]	.140
	Infant length (cm)	50 [48-51][b]	49.5 [48.5-51][b]	52 [50.25-53][a]	**.003***
	Sex				
	Female	22 (33.8%)	52 (57.2%)	12 (52.17%)	
	Male	43 (66.15%)	39 (42.8%)	11 (47.83%)	
	Siblings	28 (43.08%)	34 (37%)	10 (43.4%)	.588
	BMI z-score	0.22 [-0.52-0.86] [a]	-0.29 [-0.88-0.2] [b]	-0.37 [-1.03-0.13] [ab]	**.015***
	W/L z-score	0.26 [-0.55-0.96] [a]	-0.19 [-0.87-0.31] [b]	-0.51 [-1.52-0.16] [b]	**.002***
7 days	Infant weight (g)	3275 [2845-3585][2]	3200 [2975-3530][4]	3480 [3290-3620]	.126
	Infant length (cm)	50.75 [49-52][2b]	51 [49-51.5][4b]	52 [51-53][a]	**.028***
	Infants antibiotics	0 (0%)[2a]	7(7.7%)[3b]	0 (0%)[a]	**<.001***
	Exclusive breastfeeding	73.5%[a]	61.29%[a]	95.5%[b]	**<.001***
31 days	Infant weight (g)	4150 [3650-4728]	4100 [3685-4438] [1]	4540 [3935-4810]	.054
	Infant length (cm)	53.75 [52-55.5][b]	53.5 [52-55.5] [1b]	55.5 [54.25-57.5][a]	**.013***
	Infants antibiotics	3 (4.6%)[2]	5 (5.5%)[4]	1 (4.34%)	.922
	BMI z-score	-0.45 [-1.24-0.65]	-0.65 [-1.27-0.16][1]	-0.52 [-1.035-0.175]	.552
	W/L z-score	-0.19 [-0.89-0.55]	-0.54 [-0.54-0.34]	[-0.85- -1.275]	.104
	Exclusive breastfeeding	67.6%[a]	61.29%[a]	95.5%[b]	**<.001***
18 moths	Infant weight (Kg)	11 [10.04-11.85]	10.85 [10.85-11.54]	10.85 [9.75-11.54]	.158
	Infant length (cm)	82 [79.5-83]	81.5 [81.5-83.5]	81.5 [79-83.5]	.818
	BMI z-score	0.44 [0.22-0.58][7 a]	0.31 [0.13-0.41][7 b]	-0.13 [-0.36—0.05] [c]	**<0.001***
	W/L z-scores	0.39 [0.15-0.56][7 a]	0.26 [0.05-0.38][7 b]	-0.17 [-0.34- -0.02] [c]	**<0.001***

Normally distributed data was presented as mean ± SD and non-normal data as median [IQR]. Categorical variables were expressed as positive cases (percentage). Data no sharing letters was significantly different between studied groups. Number of missing values for each variable was represented as a superscript. Z-scores of anthropometric measures were electronically computed using WHO Anthro software (www.who.int/ childgrowth/software/en/). Z-scores values were adjusted by breastfeeding duration, antibiotic intake during the first year of life, maternal pre-gestational BMI and infant BMI and Weight for length (W/L) z-scores at birth.
