# Supplementary material for: Perinatal environment shapes microbiota colonization and infant growth: impact on host response and intestinal function
Source: Microbiome. 2020 Nov 23;8:167. doi: 10.1186/s40168-020-00940-8 (PMC7685601; doi:10.1186/s40168-020-00940-8)
Supplement: Supplementary file 3 — Additional file 2. Neonatal fecal microbiota diversity and richness of meconium and infant fecal samples at 7 and 31 days. [file 40168_2020_940_MOESM2_ESM.pdf]

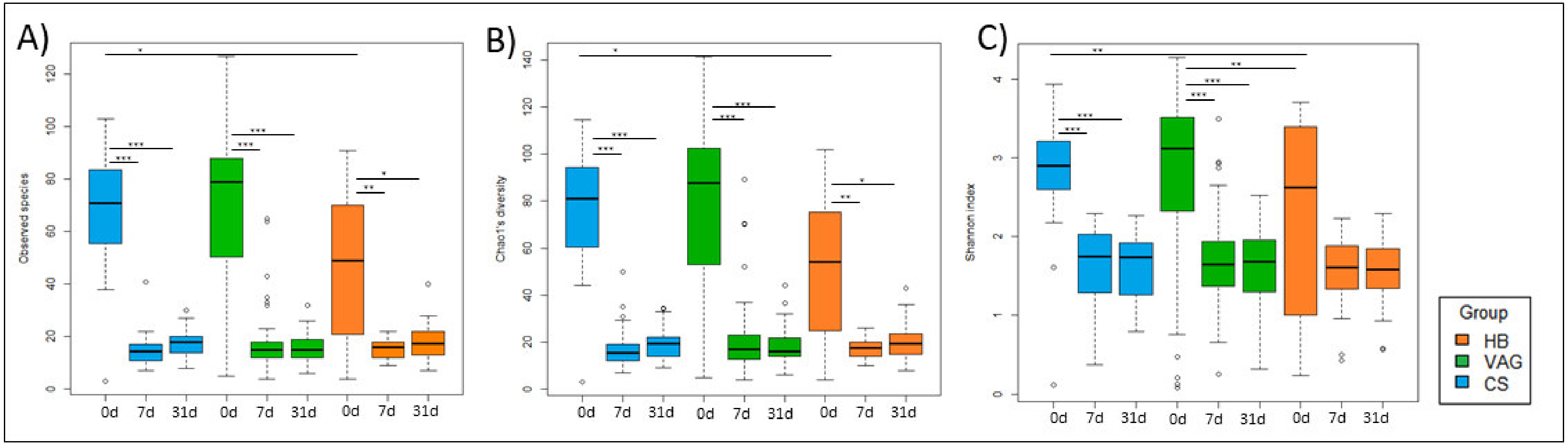

Additional file 2. Neonatal fecal microbiota diversity and richness of meconium and infant fecal samples at 7 and 31 days. Observed species (A), Chao1 (B) and Shannon (C) index were used to measure richness and diversity respectively. Samples were rarefied at 90% of the minimum depth sequencing to assess diversity. **p<0.05*, ***p<0.01*, ****p<0.001*. C-section (CS), Hospitalized vaginal delivery (VAG), homebirth (HB).
