# Supplementary material for: Perinatal environment shapes microbiota colonization and infant growth: impact on host response and intestinal function
Source: Microbiome. 2020 Nov 23;8:167. doi: 10.1186/s40168-020-00940-8 (PMC7685601; doi:10.1186/s40168-020-00940-8)
Supplement: Supplementary file 4 — Additional file 3. Relative abundance of neonatal fecal microbiota along the first month of life [file 40168_2020_940_MOESM3_ESM.pdf]

Additional file 3. Relative abundance of neonatal fecal microbiota along the first month of life

	Homebirth			Hospital Vaginal birth			C-section		
	Delivery	7d	31d	Delivery	7d	31d	Delivery	7d	31d
**Proteobacteria**	40.3a [22.8-70.2]	6.73b* [1.67-16.8]	3.06b* [1.13-12.59]	43.5a [29.2-64.75]	24.15b [8.11-39.95]	11.9b [3.95-29.63]	66.2a [35.8-77.9]	28.2b* [14.25-43.6]	24.1b* [6.19-40.4]
**Firmicutes**	43.8* [20.3-64.15]	40.6 [24.8-47.3]	26.55 [14.55-43.58]	32.8 [17.55-46.05]	36.15 [19.8-61.45]	32.2 [16.38-51.68]	19.7a* [9.27-41.1]	56.5b [38.63-76.78]	43.9b [24.4-64.1]
**Bacteroidetes**	0.25a [0.057-1.53]	0.048b [0-1.72]	0.28b [0-4.02]	2.49a [0.58-7.16]	0.1b* [0-5.13]	0.021b* [0-12.28]	0.21a [0.08-0.92]	0b* [0-0.03]	0b* [0 -0.021]
**Actinobacteria**	6.04a [1.35-11.05]	45.9b*# [27.8-53.4]	54.2b [37.73-77.3]	9.62a [3.65-15.15]	26.15a*# [0.22-48.10]	39.5b [11.87-55.43]	5.83 [2.6-8.92]	0.50 * [0.04-19.35]	14 [0.68-40]

Data was presented as median [IQR]. Data no sharing letters represent significantly different composition between times within the same delivery group. Symbols (*, #) marked differences in the microbial composition between the three studied groups at the same time.
