# Supplementary material for: Perinatal environment shapes microbiota colonization and infant growth: impact on host response and intestinal function
Source: Microbiome. 2020 Nov 23;8:167. doi: 10.1186/s40168-020-00940-8 (PMC7685601; doi:10.1186/s40168-020-00940-8)
Supplement: Supplementary file 5 — Additional file 4. Taxonomic biomarkers of microbiota composition of each group depending on place and mode of delivery. [file 40168_2020_940_MOESM4_ESM.pdf]

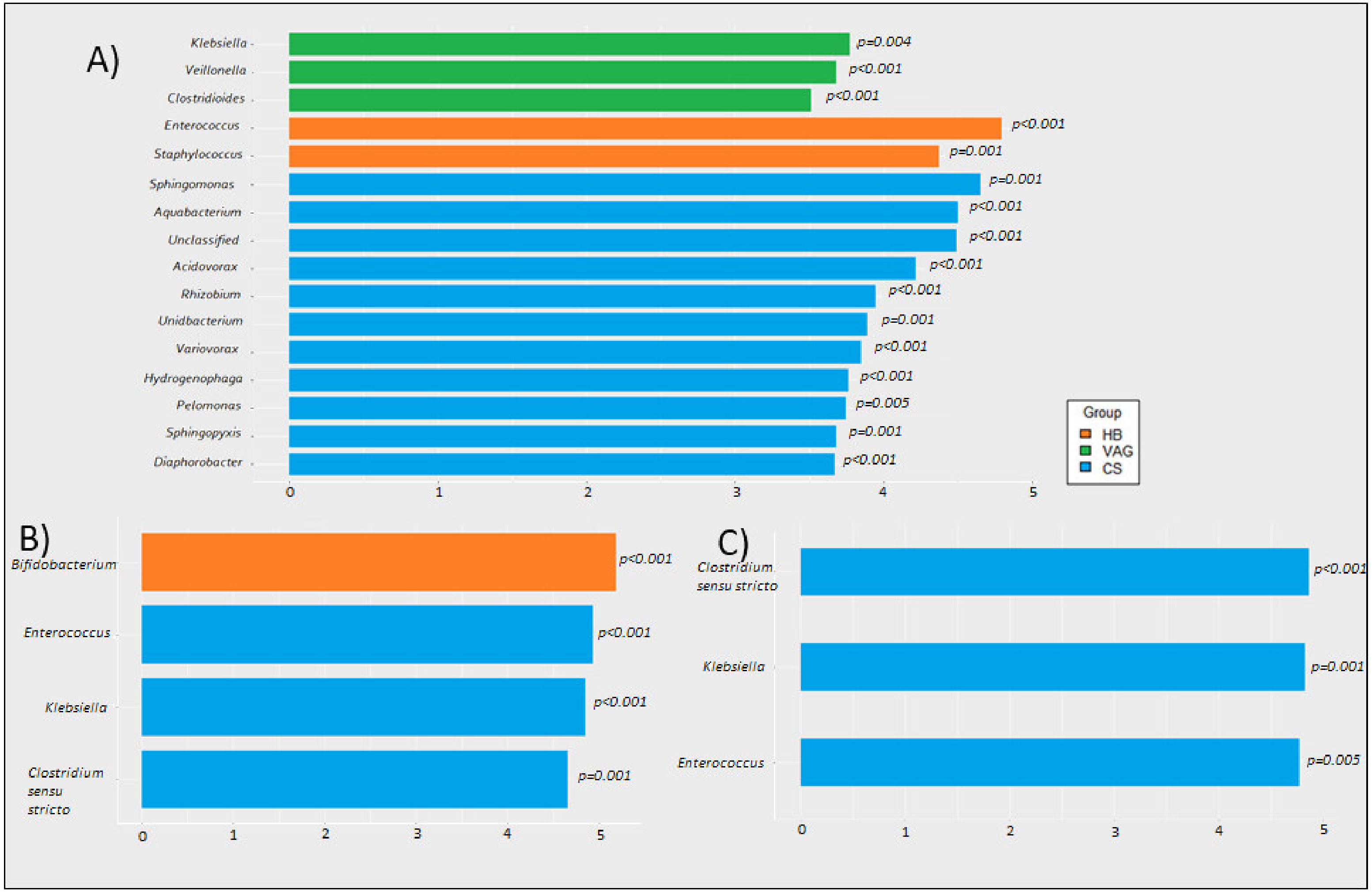

Additional file 4. Taxonomic biomarkers of microbiota composition of each group depending on place and mode of delivery. Linear discriminant analysis effect size (LEfSe) was performed with a threshold of LDA score >3 for faecal swabs microbiota at genus levels at delivery time (A), seven (B) and 31 (C) days of life.
