# Supplementary material for: Perinatal environment shapes microbiota colonization and infant growth: impact on host response and intestinal function
Source: Microbiome. 2020 Nov 23;8:167. doi: 10.1186/s40168-020-00940-8 (PMC7685601; doi:10.1186/s40168-020-00940-8)
Supplement: Supplementary file 6 — Additional file 5. Core group of neonatal microbiota composition at genus level over the first moth of life. [file 40168_2020_940_MOESM5_ESM.pdf]

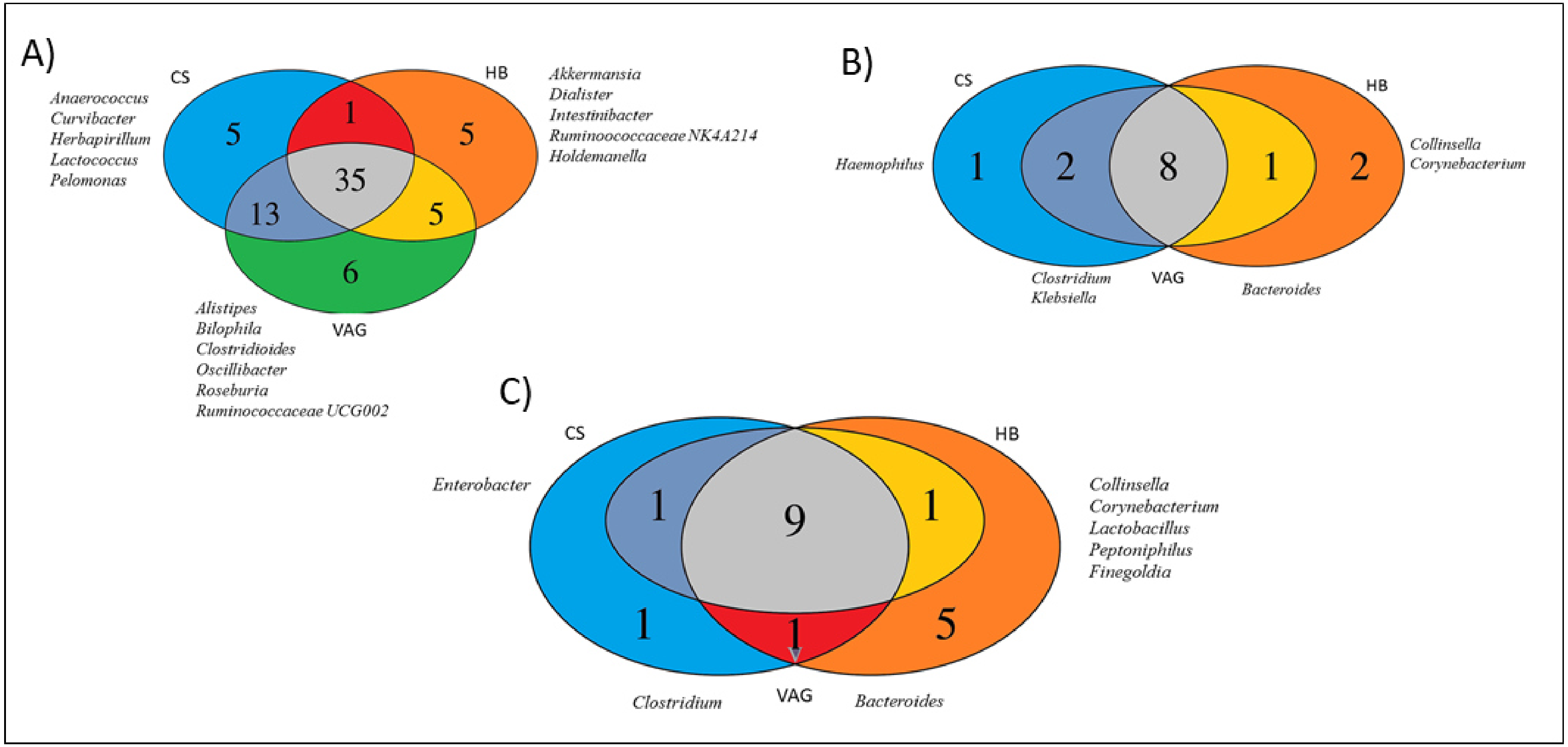

Additional file 5. Core group of neonatal microbiota composition at genus level over the first moth of life. Venn diagram was conducted at delivery (**A**), seven (**B**) and 31 days (**C**) of life.
