# Supplementary material for: Perinatal environment shapes microbiota colonization and infant growth: impact on host response and intestinal function
Source: Microbiome. 2020 Nov 23;8:167. doi: 10.1186/s40168-020-00940-8 (PMC7685601; doi:10.1186/s40168-020-00940-8)
Supplement: Supplementary file 7 — Additional file 6. Quantitative analysis of intestinal microbiota from infants born at hospital (vaginal and C-section delivery) and at home across the first month of life. [file 40168_2020_940_MOESM6_ESM.pdf]

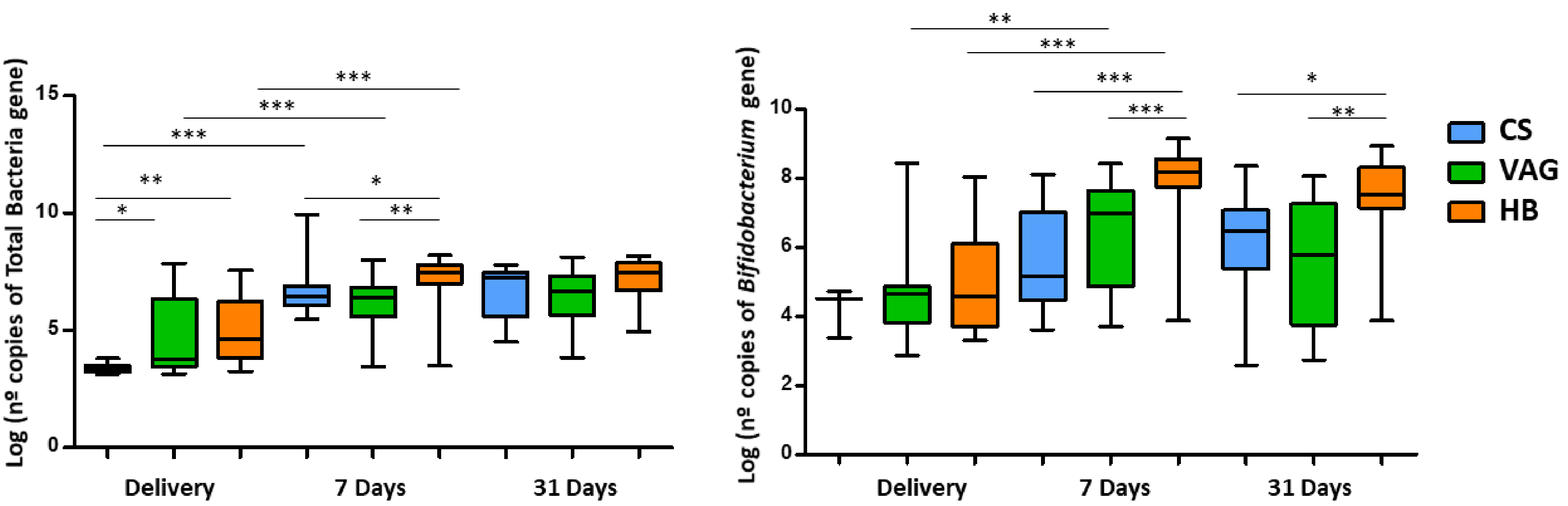

Addition file 6. Quantitative analysis of intestinal microbiota from infants born at hospital (vaginal and C-section delivery) and at home across the first month of life. Total bacterial (**A**) *and Bifidobacterium* genus counts (**B**) expressed as log (nº copies of 16S rRNA gene of each group). A subset of total population was selected according to sample availability. Percentage of samples measured according to time and groups was as follow, Delivery: VAG (47.3%), CS (69.8%), HB (95.7%); 7d: VAG (88.3%), CS (82.4%), HB (90.5%); 31d: VAG (67.2%), CS (75.8%), HB (90.9%). Data was presented as median and whiskers represented the 5-95 percentile. Kruskal-Wallis and Dunn's post hoc (FDR adjustment) test was used to test the significance of the differences in cytokine response between the groups. *$p<.05$, **$p<.01$, ***$p<.001$. C-section (CS), Hospitalized vaginal delivery (V), homebirth (HB).
