# Supplementary material for: Perinatal environment shapes microbiota colonization and infant growth: impact on host response and intestinal function
Source: Microbiome. 2020 Nov 23;8:167. doi: 10.1186/s40168-020-00940-8 (PMC7685601; doi:10.1186/s40168-020-00940-8)
Supplement: Supplementary file 8 — Additional file 7. Microbial functions related to amino acids metabolism computationally predicted present in neonatal microbiota along the first month of life. [file 40168_2020_940_MOESM7_ESM.pdf]

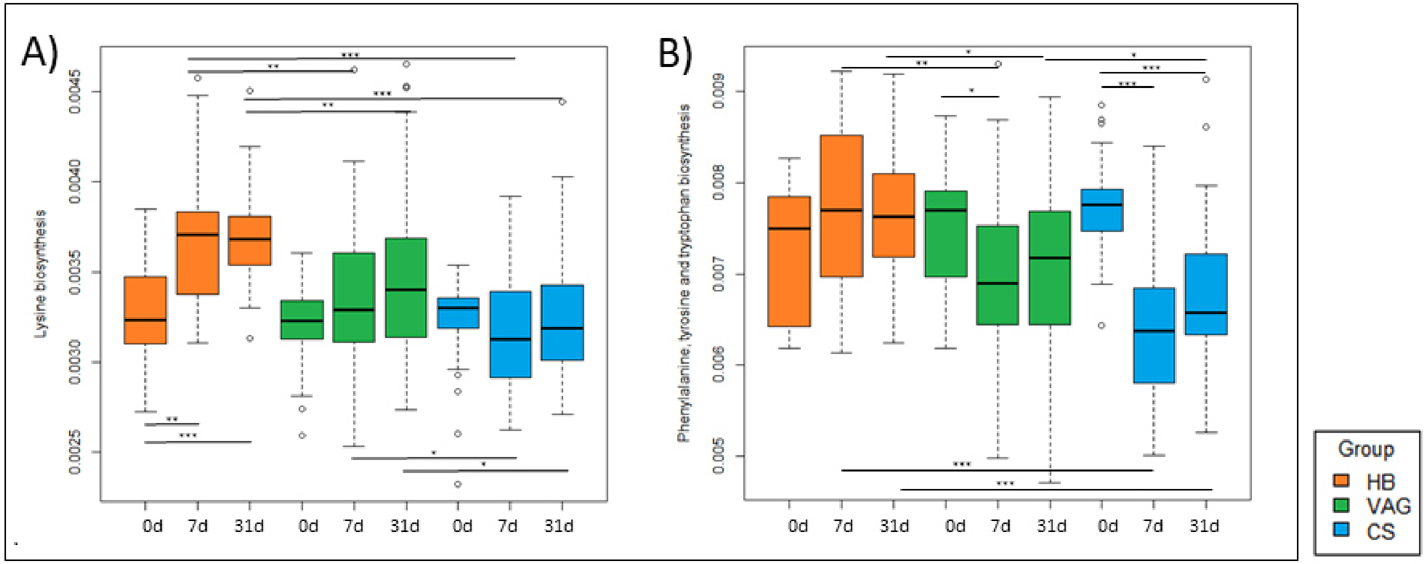

Additional file 7. Microbial functions related to amino acids metabolism computationally predicted present in neonatal microbiota along the first month of life. Computational analysis of lysine (A) and Phenylalanine, tyrosine and tryptophan byosintesis (D) routs presented in the fecal microbiota of newborns along the first month of life. Results were expressed as percentage of total functional routs for each sample. *p<.05, **p<.01, ***p<.001. C-section (CS), Hospitalized vaginal delivery (VAG), homebirth (HB).
