# Supplementary material for: Perinatal environment shapes microbiota colonization and infant growth: impact on host response and intestinal function
Source: Microbiome. 2020 Nov 23;8:167. doi: 10.1186/s40168-020-00940-8 (PMC7685601; doi:10.1186/s40168-020-00940-8)
Supplement: Supplementary file 9 — Additional file 8. Gene Expression of HT-29 and THP-1 cells after 24 h of fecal supernatant exposure. [file 40168_2020_940_MOESM8_ESM.pdf]

Additional file 8. Gene Expression of HT-29 and THP-1 cells after 24 h of fecal supernatant exposure

	HB		CS	
**Gene**	**Fold-expression**	**p-value**	**Fold expression**	**p-value**
**HT-29 cells**				
HP	0.202 (0.088 - 0.478)	***< 0.001**** ⬇	0.265 (0.098 - 0.726)	***0.006**** ⬇
CDH1	0.226 (0.065 - 0.704)	***0.005**** ⬇	0.326 (0.102 - 1.129)	***0.035**** ⬇
OCLN	0.192 (0.080 - 0.485)	***<0 .001**** ⬇	0.253 (0.088 - 0.739)	***0.012**** ⬇
TLR4	1.405 (1.052 - 1.735)	***0.024**** ⬆	1.482 (1.115 - 2.052)	*0.100*
IRAK4	2.654 (1.216 - 4.330)	***<0 .001**** ⬆	1.965 (0.952 - 4.218)	*0.174*
IL12	0.609 (0.311 - 1.036)	*0.118*	0.725 (0.435 - 1.310)	*0.210*
CXCL10	989.712 (654.81 - 1637.46)	***< 0.001**** ⬆	677.505 (214.19 - 1584.86)	***0.033**** ⬆
FOS	1.029 (0.796 - 1.244)	*0.814*	1.333 (0.847 - 2.046)	*0.210*
**THP-1 cells**				
TLR4	0.917 (0.342 - 12.306)	*1*	0.509 (0.345 - 0.626)	***0.006**** ⬇
IRAK4	1.345 (0.399 - 45.200)	*0.969*	0.538 (0.209 - 0.959)	*0.113*
IL12	0.418 (0.110 - 16.175)	*0.599*	8.437 (0.831 - 107,927.33)	*0.379*
CXCL10	1751.59 (104.706 - 197,216.41)	***< 0.001**** ⬆	893.79 (652.843 - 1295.368)	***<0.001**** ⬆
FOS	0 (0.000 - 0.233)	*0.271*	0.238 (0.097 - 0.628	***0.038**** ⬇

Total RNA was extracted from cells treated with homebirth (HB) samples and C-section (CS) fecal supernatant. Values expressed relative expression fold-change of each condition compared to control (95% C.I). *P<.05* (*) and blond letters marked significant differences between treatment and control, up (UP) or down (DOWN) regulation was represented by the arrows.
