# Supplementary material for: Perinatal environment shapes microbiota colonization and infant growth: impact on host response and intestinal function
Source: Microbiome. 2020 Nov 23;8:167. doi: 10.1186/s40168-020-00940-8 (PMC7685601; doi:10.1186/s40168-020-00940-8)
Supplement: Supplementary file 10 — Additional file 9. Epithelial barrier function and maturation of simulated intestinal epithelium of the triple co-culture during the long-term exposure [file 40168_2020_940_MOESM9_ESM.pdf]

Additional file 9. Epithelial barrier function and maturation of simulated intestinal epithelium of the triple co-culture during the long-term exposure

		HB	VAG	CS	
**Trans-epithelial electrical resistance (TEER) - Ohms/cm^2**					**p-value**
**Day**	**1**	666.02 ± 12.06	652.41 ± 29.97	643.10 ± 6.66	*0.226*
	**2**	735.09 ± 40.76	831.83 ± 62.11	782.08 ± 43.62	*0.211*
	**3**	763.20 ± 111.65	848.98 ± 105.23	838.91 ± 76.81	*0.624*
	**4**	555.72 ± 63.47	732.92 ± 220.33	689.30 ± 246.44	*0.198*
	**5**	556.10 ± 17.99	638.29 ± 77.09	642.19 ± 209.07	*0.350*
	**6**	682.83 ± 65.45[a]	865.44 ± 86.64[b]	822.35 ± 47.32 [b]	***0.001****
	**7**	903.66 ± 72.439[a]	727.81 ± 101.87[b]	732.35 ± 56.60[b]	***0.004****
**Apparent permeability – cm/s**					
		$1.45{\cdot}10^{-6} \pm 1.72{\cdot}10^{-7}$[a]	$2.09{\cdot}10^{-6} \pm 5.68{\cdot}10^{-7}$[b]	$2.01{\cdot}10^{-6} \pm 3.49{\cdot}10^{-7}$[b]	***0.013****
**Intestinal alkaline phosphatase – mg/ml**					
**Day**	**1**	0.56 ± 0.2	0.40 ± 0.13	0.42 ± 0.12	*0.106*
	**2**	0.75 ± 0.11[a]	0.48 ± 0.21[b]	0.47 ± 0.13[b]	***0.023****
	**4**	1.02 ± 0.46	1.02 ± 0.38	1.01 ± 0.34	*0.997*
	**6**	0.85 ± 0.28	0.68 ± 0.32	0.69 ± 0.27	*0.494*
	**7**	0.75 ± 0.24[a]	0.42 ± 0.24[b]	0.43 ± 0.32[b]	***0.027****
**Mucus production – mg/ml**					
		0.691 ± 0.09	0.928 ± 0.25	0.920 ± 0.28	*0.107*

Variations in the Transepithelial electrical resistance (TEER) and intestinal alkaline phosphatase were presented along the seven days of treatment. Apparent permeability and mucus production were measured at final time point. Letters indicated groups that showed significant differences between them within a measurable variable. *P<0.05* (*) and blond letters marked significant differences between three studied groups.
