# Supplementary material for: Perinatal environment shapes microbiota colonization and infant growth: impact on host response and intestinal function
Source: Microbiome. 2020 Nov 23;8:167. doi: 10.1186/s40168-020-00940-8 (PMC7685601; doi:10.1186/s40168-020-00940-8)
Supplement: Supplementary file 11 — Additional file 10. Flow chart of study participants. [file 40168_2020_940_MOESM10_ESM.pdf]

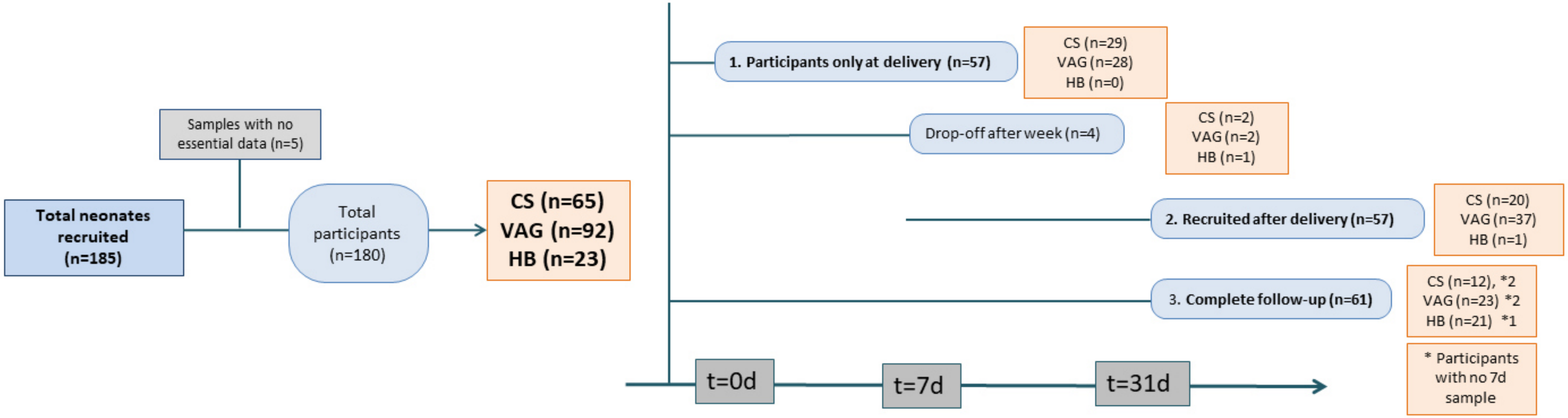

Additional file 10. Flowchart detailing the number of participants in each time point according to study group. The three main participation mode of MAMI cohort [71] is shown in bold letters. Mode 1 (Participants only at delivery time), Mode 2 (Participants recruited in health care centers after delivery time), Mode 3 (Participants recruited at hospital before delivery and follow in the study during the first month of life). Additionally it is also shown the drop-off of some participants according to study group (n=4) and number of participants with missing samples at 7d (n=6).
