# Supplementary material for: Perinatal environment shapes microbiota colonization and infant growth: impact on host response and intestinal function
Source: Microbiome. 2020 Nov 23;8:167. doi: 10.1186/s40168-020-00940-8 (PMC7685601; doi:10.1186/s40168-020-00940-8)
Supplement: Supplementary file 13 — Additional file 12. Primers of 16 s rRNA gene of prokaryotic targets and human genes tested by qPCR. [file 40168_2020_940_MOESM12_ESM.pdf]

Additional file 12. Primers of 16s rRNA gene of procariotic targets and human genes tested by RT-PCR.

Target	Sequence (5'-3')		Amplicon size (bp)	Reference
**PROCARIOTIC PRIMERS**		**Annealing (C°)**		
**Total Bacteria**	CGTGCCAGCAGCCGCGG<br>TGGACTACCAGGGTATCTAATCCTG	62	274	[1,2]
Bifidobacterium	GATTCTGGCTCAGGATGAACGC<br>CTGATAGGACGCGACCCCAT	60	232	[3]
**HUMAN GENES PRIMERS**		**Efficiency at 58ºC**		
**ACTB**	ATGCTATCACCTCCCCTGTGTG<br>TTGTTACAGGAAGTCCCTTGCC	1.79	101	[4]
**TLR2**	CTGGTAGTTGTGGGTTGAAGCA<br>GATTGGAGGATTCTTCCTTGGA	1.71	102	[5]
**TLR3**	ACCTCAACTGGGATCTCGTCA<br>ACAACTTAGCACGGCTCTGGA	1.82	124	[6]
**TLR4**	GGGTTCAGGGACAGGTCTAAAGA<br>AATCTAGAGCACTTGGACCTTTCC	1.81	116	[6]
**TOLLIP**	AAGTGGTACAGCCTGAGCGG<br>GATGGGCACATAGCCAACGC	1.75	159	This work
**IRAK4**	CGCCCGGGCAGGAATAGAA<br>TTCAGAAGTGGGACTTTTTCCAGT	1.79	227	This work
**FOS**	CAGACTACGAGGCGTCATCC<br>CGTGGGAATGAAGTTGGCAC	1.67	166	[7]
**IP10 (CXCL10)**	GTGGCATTCAAGGAGTACCTC<br>GCCTTCGATTCTGGATTCAGACA	1.77	193	[8]
**IL12**	TTGTGGCTACCCTGGTCCT<br>AGAGTTTGTCTGGCCTTCTGG	1.75	151	[9]
**IL10**	GGCGCTGTCATCGATTTCTT<br>TGGAGCTTATTAAAGGCATTCTTCA	1.77	79	[10]
**ZO-1 (HP)**	TTAAGC-CAGCCTCTCAACAGAAA<br>GGTTGATGATGCTGGGTTTGT	1.75	83	[11]
**OCLN**	TATAAATCCACGCCGGTTCCT<br>ACGAGGCTGCCTGAAGTCAT	1.78	77	[11]
**ECAD (CDH1)**	ACAGCCCCGCCTTATGATT<br>TCGGAACCGCTTCCTTCA	1.79	60	[12]
**HPRT1**	TGACACTGGCAAAACAATGCA<br>GGTCCTTTTCACCAGCAAGCT	1.80	93	[13]

REFERENCES

1. Caporaso JG, Lauber CL, Walters WA, Berg-Lyons D, Lozupone CA, Turnbaugh PJ, et al. Global patterns of 16S rRNA diversity at a depth of millions of sequences per sample. Proc Natl Acad Sci U S A. National Academy of Sciences; 2011;108:4516–22.

2. Cruaud P, Vigneron A, Lucchetti-Miganeh C, Ciron PE, Godfroy A, Cambon-Bonavita MA. Influence of DNA extraction method, 16S rRNA targeted hypervariable regions, and sample origin on microbial diversity detected by 454 pyrosequencing in marine chemosynthetic ecosystems. Appl Environ Microbiol. American Society for Microbiology; 2014;80:4626–39.

3. Gueimonde M, Tölkkö S, Korpimäki T, Salminen S. New real-time quantitative PCR procedure for quantification of bifidobacteria in human fecal samples. Appl Environ Microbiol. American Society for Microbiology (ASM); 2004;70:4165–9.

4. Pattyn F. RTPrimerDB: the real-time PCR primer and probe database, major update 2006. Nucleic Acids Res. Oxford University Press (OUP); 2006;34:D684–8.

5. Romieu-Mourez R, François M, Boivin M-N, Bouchentouf M, Spaner DE, Galipeau J. Cytokine Modulation of TLR Expression and Activation in Mesenchymal Stromal Cells Leads to a Proinflammatory Phenotype. J Immunol. The American Association of Immunologists; 2009;182:7963–73.

6. Chen Z, Cheng Y, Xu Y, Liao J, Zhang X, Hu Y, et al. Expression profiles and function of Toll-like receptors 2 and 4 in peripheral blood mononuclear cells of chronic hepatitis B patients. Clin Immunol. 2008;128:400–8.

7. Gimeno-Alcañiz J V, Collado MC. Impact of human milk on the transcriptomic response of fetal intestinal epithelial cells reveals expression changes of immune-related genes. Food Funct. 2019;10:140.

8. Wang X, Seed B. Selection of oligonucleotide probes for protein coding sequences. Bioinformatics. 2003;19:796–802.

9. Zenhom M, Hyder A, De Vrese M, Heller KJ, Roeder T, Schrezenmeir J. Peptidoglycan recognition protein 3 (PglyRP3) has an anti-inflammatory role in intestinal epithelial cells. Immunobiology. Urban & Fischer; 2012;217:412–9.

10. Wang YC, Sung WW, Wu TC, Wang L, Chien WP, Cheng YW, et al. Interleukin-10 haplotype may predict survival and relapse in resected non-small cell lung cancer. PLoS One. Public Library of Science; 2012;7.

11. Drago S, El Asmar R, Di Pierro M, Clemente MG, Tripathi A, Sapone A, et al. Gliadin, zonulin and gut permeability: Effects on celiac and non-celiac intestinal mucosa and intestinal cell lines. Scand J Gastroenterol. 2006;41:408–19.

12. Massa F, Devader C, Lacas-Gervais S, Béraud-Dufour S, Coppola T, Mazella J. Impairement of HT29 cancer cells cohesion by the soluble form of neurotensin receptor-3. Genes and Cancer. Impact Journals LLC; 2014;5:240–9.

13. Vandesompele J, De Preter K, Pattyn F, Poppe B, Van Roy N, De Paepe A, et al. Accurate normalization of real-time quantitative RT-PCR data by geometric averaging of multiple internal control genes. Genome Biol. BioMed Central; 2002;3:research0034.1.
